# The impact of Ukraine’s war outbreak on green preferences in Europe

**DOI:** 10.1007/s13280-025-02173-1

**Published:** 2025-04-22

**Authors:** Enrico Angelo Raffaele D’Ecclesiis, Eugenio Levi, Fabrizio Patriarca

**Affiliations:** 1 Department of Economics, University of Modena and Reggio Emilia, Via Jacopo Berengario, 51, 41121 Modena, Italy; 2 Department of Human Sciences, Link Campus University, Via Casale S. Pio V, 44, 00165 Rome, Italy

**Keywords:** Climate change, Environmental preferences, War

## Abstract

This study examines the impact of the Ukraine war on pro-environmental attitudes. Using the 2021–2022 wave of the European Social Survey, we leverage the timing of the war’s outbreak in February 2022 as a natural experiment. By comparing the pro-environmental preferences of individuals interviewed just before and after the war’s onset across nine European countries, we find a significant decline on political preferences for pro-environmental political parties and concerns for climate change. Further investigation reveals that rising energy prices and the salience of the war itself, as measured by Google searches, partially mediate these effects.

**Supplementary Information:**

The online version contains supplementary material available at 10.1007/s13280-025-02173-1.

## Introduction

The war between Ukraine and Russia may have marked a shift in the way Europeans see environmental issues. The problematic future of the soon-to-be-implemented European Green Deal makes this issue especially compelling. Before the war, pro-environmental attitudes were gaining ground^1^, while now the level of economic and policy uncertainty has risen and other issues, like energy prices and the war itself, may have become more salient. Furthermore, the Green political parties were not able to repeat the impressive outcome of the 2019 European elections, when they were able to reach 10% of the votes. There was a reversion to the mean with the recent 2024 European Parliamentary elections, when especially the German and French Green parties–the largest and oldest ones in Europe–almost halved their votes. We empirically test in this paper if the war indeed negatively impacted pro-environmental preferences; we find that it did.

So far, many papers have discussed the determinants of pro-environmental support (see Drews and Van den Bergh 2016 for a review) and of voting for Green political parties (Schumacher 2014; Grant and Tilley 2019; Hoffmann et al. 2022). However, to the best of our knowledge, an investigation on how conflicts directly influence pro-environmental support is still missing. Conflicts seem to increase in-group cooperation (e.g., Voors et al. 2012; Bauer et al. 2016), which could potentially lead to more pro-environmental support. This evidence considers the influence of conflicts over the same population that underwent the conflict, which is not the case for Europe in the war in Ukraine though. The Ukraine war may be considered more akin to a period of financial distress for Europe, due to heightened uncertainty. On this, while the effect of recessions on pro-environmental preferences is sometimes negative (Kenny 2020; Meyer 2022; Hartmann and Preisendörfer 2024), some studies find a null effect (e.g., Kenny 2024). Alternatively, the war may have changed the relative salience of the political issues, increasing the salience of the war itself at the expense of all other not directly related issues, i.e., among them the environment (Ansolabehere and Iyengar 1994; Bromley-Trujillo and Poe 2020; Bordalo et al. 2022).

In the Ukraine war, we consider the timing of the outburst of the war, February 24, 2022, as a natural experiment. In fact, while since the end of 2021, there was a partial sense in the public opinion that the war could break out, the specific timing was unknown to everyone, including the public opinion of Russia and Ukraine. We use the European Social Survey (ESS) data from the 2022 wave and exploit the date of the interviews: in a nutshell, we compare pro-environmental preferences of individuals just before and after the outburst of the war within each country for which the timing of the survey fielding works (Belgium, Switzerland, Spain, the UK, Greece, Italy, the Netherlands, Norway, Poland). We run regressions that control for country fixed effects with an event study approach first, then in a reduced form framework. Both approaches confirm that the war in Ukraine reduced both closeness to pro-environmental political parties and concerns about climate change, while the effect is not significant on personal responsibility over climate change. Further analyses suggest that these effects are partly mediated by energy prices and the salience of the war, measured with “Ukraine” Google searches.

## Materials and methods

### Data sources and preparation

The main data source is the ESS, a well-established biennial European level survey. Wave 10 covers the period of interest around the war’s outbreak, wave 8 and 9 are used to estimate monthly trends control variables. The four dependent variables cover several dimensions of attitudes toward the environment and policy preferences:

- *Climate change worry:* a sentiment variable indicating people’s concerns about climate change.
- *Personal responsibility:* a measure for the personal responsibility to reduce climate change.
- *Environmental salience*: a political variable ranking the party to which individuals feel closest, based on the salience of ecology and sustainability issues in its statements.
- *Environmental position*: a political variable ranking the party to which individuals feel closest, based on its positions on environmental protection and sustainability, even at the cost of sacrificing economic growth.

To obtain the first two variables, we consider the questions from the ESS “How worried about climate change” and “To what extent do you feel personal responsibility to reduce climate change”, respectively, both of them originally defined on a Likert scale. To obtain the political variables, we consider the answer to the question “Which party feel close to” and match it with the party classifications from the Chapel Hill Expert Survey (CHES) about “importance/salience of environmental sustainability” ranging from “0 – not important at all” and“10 – extremely important”, and the classification about “position toward environmental sustainability” ranging from “0 – strongly supports environmental protection even at the cost of economic growth” and “10 – strongly supports economic growth even at the cost of environmental protection” (reverted). For greater comparability of the results across the different outcomes, we standardized the four dependent variables.

The analysis includes the countries that are both in the ESS and in the CHES database, among which we exclude countries who had more than 90% interviews before war’s outbreak or more than 90% after. Accordingly, the analysis covers Belgium, Switzerland, Spain, UK, Greece, Italy, Netherlands, Norway, and Poland. Main control variables are also drawn from the ESS: a dummy distinguishing between rural and urban areas, age groups, gender, educational level from ISCED classification, main activity performed in the last 7 days, and perceived income. Descriptive statistics on these variables are shown in Supplementary Information Appendix S1.

In further analyses, we also consider energy prices and the frequencies of weekly Google searches for the term “Ukraine" at country level from Google Trends. For energy prices, we rely on data of the national bidding zones of the European Network of Transmission System Operators for Electricity (ENTSO-E)^2^. Energy prices have been considered in log and in their monthly average. Since perception of prices can differ from actual prices, we considered a 1-month lag to allow energy prices to reflect in consumer perceptions^3^.

### Methodology

**Fig. 1:**
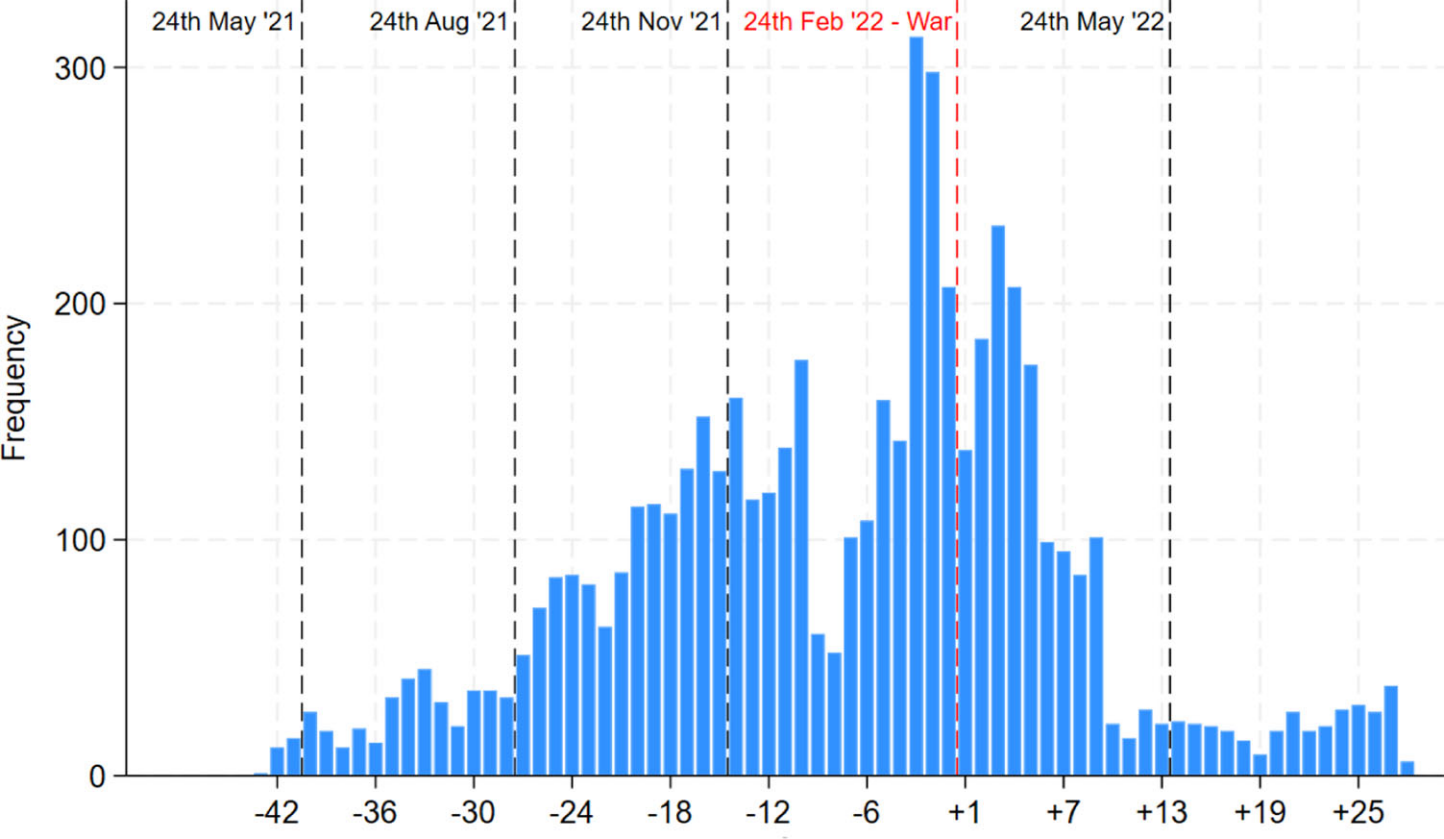
Weekly temporal distribution of interview completion dates. Vertical lines mark the time boundaries of sub-periods in the event study

**Fig. 2:**
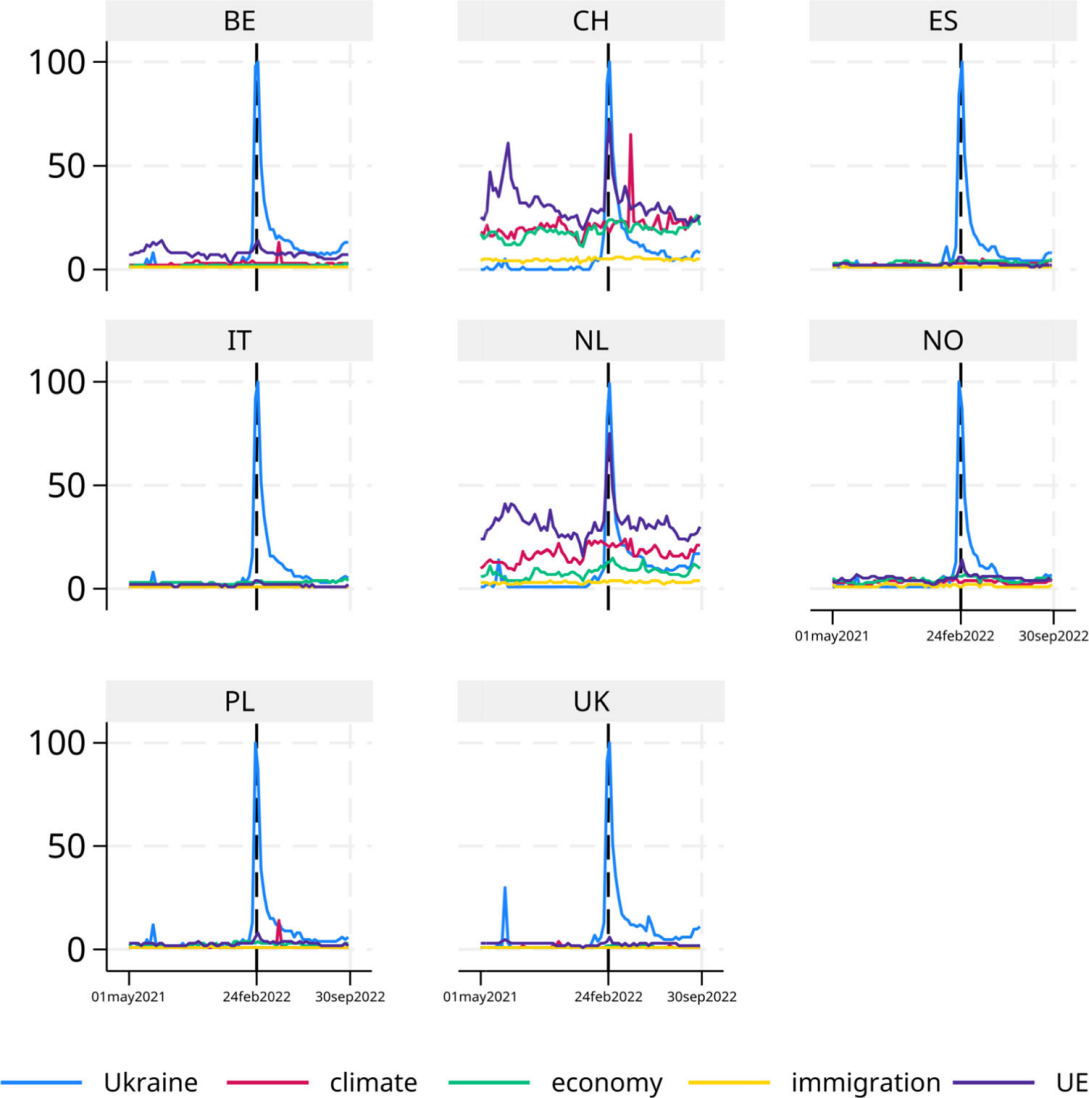
Google searches over several topics. In each panel weekly Google searches on Ukraine or on several topics (climate, the economy, immigration, and UE) are represented for a specific country (Belgium, Switzerland, Spain, Italy, the Netherlands, Norway, Poland, and the UK). The vertical line marks the February 24, 2022, the outbreak of the war

The identification strategy relies on the availability of interview dates, most of which were conducted around the outbreak of the war (Figure 1). This information is used to perform both an event study and a broader pre-post analysis. For the event study, we employed quarterly intervals–the shortest span that ensured a sufficient number of observations in each time period. This approach covers an entire calendar year. In our framework, the control group consists of individuals interviewed in the quarter preceding the war’s outbreak, specifically between November 21, 2021, and February 24, 2022. This methodology aligns with previous studies that have examined the impacts of nationwide or international events in the absence of a contemporaneous control group (e.g., Garcia 2013; Szeidl and Szucs 2021). A standard assumption for identification is the exogeneity of the event’s timing, which is supported in our case by multiple accounts of the invasion. The news reports from those days and recent recounts of the invasion in books (e.g., Kostyuchenko et al. 2024) are very clear on this point. It seems that even high officials in Moscow and Kiev were unaware of the specific starting date of the invasion. We look at Google searches for several topics (climate change, the economy, immigration, and the European Union) alongside “Ukraine" in all the countries under analysis and find that there was an abrupt spike in searches for “Ukraine" in the week of the outbreak in each country with no pre-trends (see Figure 2). Furthermore, we do not see spikes on the other topics if not for a moderate spike on searches related to the European Union in some countries–probably related to its response to the war–suggesting that in the quarter under consideration no other major event happened that was as relevant as the war. Additionally, we employ several methods and robustness tests to ensure that our results are not influenced by seasonal effects or long-term trends in pro-environmental attitudes. For the pre-post analysis, we use a dummy variable that equals zero before February 24, 2022, and one thereafter.

The model specification that we estimate with OLS is:

1 $$\begin{aligned}&y^j_i=\alpha ^j+\gamma ^{j}T_i+\pmb {\beta }^{j}{\textbf{X}}_i + c^j_i + \epsilon ^j_i \end{aligned}$$

where $j \in (1,4)$ identifies the specific dependent variable of interest, and *i* represents the individual. ${\textbf{X}}_i$ denotes the vector of controls, $c^j_i$ represents the country fixed effects, and $\epsilon ^j_i$ is the error term. The coefficient of interest, $\gamma ^j$, is associated with the variable $T_i$, which indicates whether the individual was interviewed before or after the war’s outbreak, or in the case of the event study, during specific three-month intervals. To address potential seasonality effects or time trends in the event study, we include in ${\textbf{X}}_i$ a variable derived from the coefficients of month fixed effects obtained from regressions on the same dependent variables using previous ESS waves since 2016 (see Table S2 - 7 for these regression results). Controls further include gender, living in a big city, income, education, age groups, and main activity. To account for differences in individuals’ characteristics (including country-level variations) across periods, we apply a weighting approach using the inverse probability weighting (IPW) method. For the event studies, the IPW weights are based on the sample composition of the benchmark quarter, while for the pre–post-regressions, they are based on the pre-war period. Standard errors are robust.

## Results

**Fig. 3:**
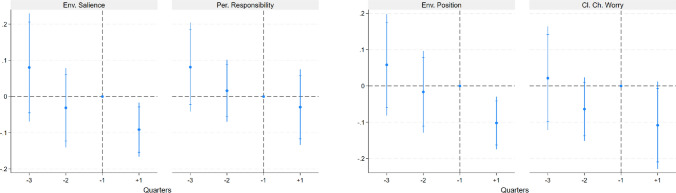
Event Study. Event graphs come from OLS regressions on 5026 observations on standardized variables over closeness to parties with a strong focus on the environment, personal responsibility toward climate change, closeness to pro-environmental parties, and concerns about climate change. Controls include gender, living in a big city, income, education, age groups, main activity, a control for monthly trends, and country fixed effects. Independent variables of interest are quarters with November 24–February 23, 2022 (-1) as the benchmark. Confidence intervals are at 90 and 95%. The full results from the regression can be found in Table  S2 - 1

Figure 3 presents the results of the event study for the four pro-environmental measures described above. The analysis includes three quarters before and one quarter after the outbreak of the war, using the quarter immediately preceding the outbreak (−1) as the benchmark and excluding sparse data from periods outside this range (see Fig. 1). The results reveal a negative and significant impact of the war on variables related to closeness to political parties-both when these parties are ranked by the salience of environmental issues in their platforms and by the greenness of their proposed policies–as well as on the variable measuring concerns about climate change. These effects are substantial, corresponding to decreases of 9.51, 9.95, and 10.20% of a standard deviation, respectively. In contrast, the measure of personal responsibility remains unaffected.

**Table 1:** OLS regressions on pro-environmental preferences. OLS regressions on 5408 observations on standardized variables over closeness to parties with a strong focus on the environment, personal responsibility toward climate change, closeness to pro-environmental parties, and concerns for climate change. Each panel shows results of a separate regression, where the independent variables of interest are either Post-war’s outbreak, Energy prices, or GTrends on “Ukraine”, Post together with, alternatively, the other two. Controls include gender, living in a big city, income, education, age groups, main activity, and country fixed effects. The full results of the regressions can be found from Table  S2 - 2 to S2 - 6. *** p < 0.01, ** p < 0.05, * p < 0.10

	Env. Sal.	Env. Pos.	Per. Resp.	Worry
Post	−0.0993***	−0.0930***	−0.0401	−0.0884***
	(0.0332)	(0.0331)	(0.0321)	(0.0331)
Energy	−0.1922***	−0.1699**	−0.1295**	−0.0851
	(0.0663)	(0.0694)	(0.0555)	(0.0634)
GTrends	−0.0016**	−0.0013*	−0.001	−0.0014**
	(0.0006)	(0.0007)	(0.0006)	(0.0007)
Post	−0.0715**	−0.0692**	−0.0172	−0.0829**
	(0.0322)	(0.0328)	(0.0329)	(0.0333)
Energy	−0.1434**	−0.1227*	−0.1178**	−0.0286
	(0.0691)	(0.0666)	(0.0565)	(0.0640)
Post	−0.0761*	−0.0775*	−0.015	−0.0683*
	(0.041)	(0.0404)	(0.0392)	(0.0406)
GTrends	−0.0008	−0.0005	−0.0009	−0.0007
	(0.0008)	(0.0009)	(0.0008)	(0.0009)

In Table 1, we present the coefficients of interest from the OLS estimations, where the time dimension is reduced to two periods (pre- and post-war outbreak). Additionally, we incorporate energy prices in the local bidding zones (lagged by 1 month) and Google search trends for the term "Ukraine" from Google Trends in separate models (see the Supplementary Information for further details).

The first model reaffirms the findings of the event study. When considered individually in the same model, both Google searches and energy prices are significantly correlated with environmental preferences. In the final models, we examine how much the war’s impact is mediated by these two variables by assessing the reduction in the pre–post-war coefficient when they are separately included as controls. Our results indicate that the war’s effect is partially mediated by rising energy prices and, to a lesser extent, by the salience of the war. Depending on the outcome variable, the pre–post-war coefficient decreases by 7 to 25%. Notably, the energy price channel exhibits an independent effect beyond the war event, as its associated coefficient is significant in all regressions except for the one concerning climate change concerns.

**Fig. 4:**
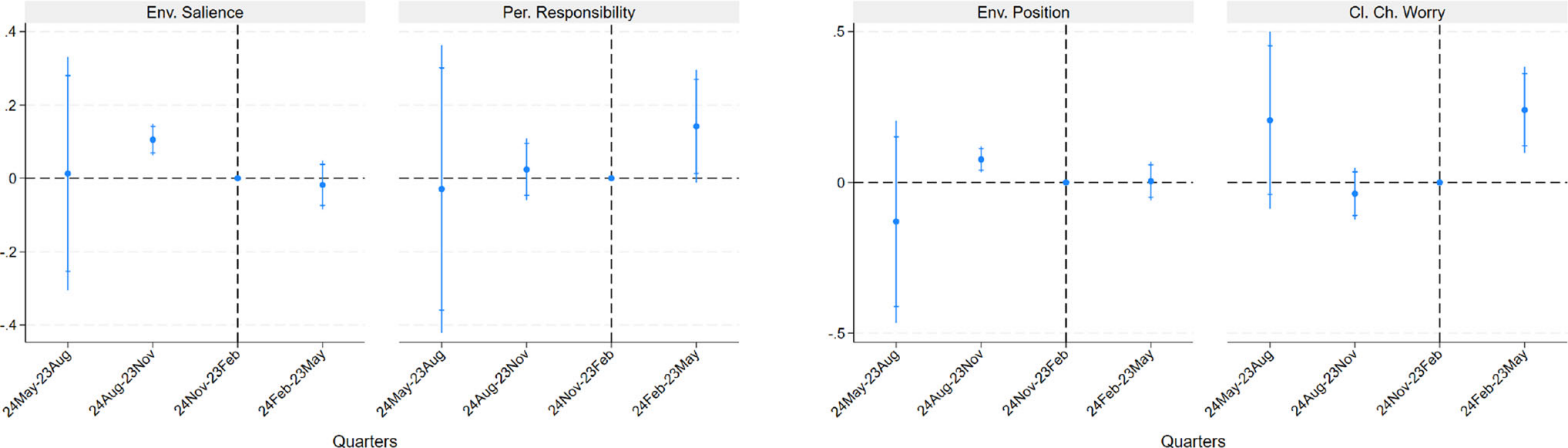
Placebo event study on 2016-2019. Event graphs come from OLS regressions on 12640 observations on standardized variables over closeness to parties with a strong focus on the environment, personal responsibility toward climate change, closeness to pro-environmental parties, and concerns about climate change. The sample for Pers. Responsibility and Cl. Ch. Worry is restricted to 2016 and 2017 (6231 observations) because of data availability. Controls include gender, living in a big city, income, education, age groups, main activity, a control for a year trend, and country fixed effects. Independent variables of interest are quarters with November 24–February 23 (-1) as the benchmark. Confidence intervals are at 90 and 95%. Results from the full regression are shown in Table S2 - 8

Our regressions already control for short-time trends because of the inclusion in the event study of the variable that incorporates month fixed effects from 2016 to 2017 as control. However, to check that our results are not driven by long-time declining trends in pro-environmental attitudes, we perform a placebo test where the same model is applied to ESS data from 2016 to 2019^4^. The event study is represented in Figure 4 and it shows that, if anything, closeness to pro-environmental parties in a normal year seems to be higher in the quarter before the benchmark, that is between September and November. As a further check, we explore long-time trends in all our outcome variables by considering an extended time period between 2016 and 2022 (either with or without 2020, the year when COVID exploded) and testing in our baseline model for the presence of a trend at daily level (see Tables S4 - 1 and S4 - 2). We find a strongly significant and positive trend in all our outcomes, which goes counter the idea that our results may be driven by a long-term declining trend, and is more consistent with the hypothesis that pro-environmental preferences are slowly increasing over time due to increased awareness of the dangers of climate change.

These results are robust to several additional tests detailed in the Appendix S5. In all tests, we add an alternative variable to personal responsibility representing individual sensitivity to the environment to confirm that there are no effects of the war on policy-unrelated variables. First, we exclude IPW weights (Table S5 - 1 and S5 - 2), the control for seasonality (Table S5 - 3) or both (Table S5 - 4). Then, we consider alternative lag specifications for energy prices (Tables S5 - 5, S5 - 6 and S5 - 7). Finally, we use a leave-one-out validation to check that no specific country is driving our results (from Table  S5 - 8 to S5 - 16). All results are robust to these tests and results on sensitivity confirm that the war had no clear effect on policy-unrelated outcomes. A specific concern could be that the timing of the interviews is correlated with political orientation and interest into politics; this is why we reproduce the event study with the inclusion of these two additional controls^5^ (see Figure S5 - 1). Unsurprisingly, we find that left-wing individuals and individuals who are more interested into politics are more pro-environmental. However, the results related to the effect of the war are indistinguishable from our main results, which not only alleviates this concern but also suggests that these two political variables are not in the causal chain from war to reduced pro-environmental attitudes.

## Discussion

The outbreak of Ukraine war reduced pro-environmental preferences. The war in Gaza and further uncertainty related to the electoral outcome in the US may render our results even more relevant now than when the war broke out. Unfortunately, we cannot really study the persistence of the effect in our setup because we cannot use data from after August. This is because the ESS follows a cyclical pattern in fielding the survey by country, meaning that in each country, interviews are concentrated in a limited time period. Interviews fielded after August 2022 would not be comparable to the pre-outburst period anymore, as they would be coming from different countries altogether. This is why we leave an investigation of the medium-run effect of the war for future research.

Our result, especially in the light of the moderating role of energy prices, seems at first sight in line with previous evidence that materialistic concerns crowd out concerns about the environment. However, the mediating role of Google searches suggests a further channel related to the reduced salience of environmental issues after the war. Consistently with political science and political economy literature, a shift in issue salience may have brought about a corresponding change in environmental political preferences ( Bélanger and Meguid 2008: Ansolabehere and Puy 2018: Bromley-Trujillo and Poe 2020). Besides, by additional heterogeneity analyses on political opinions, we find that the effect of the war is negative regardless of income or education (see Tables S3 - 1 and S3 - 2). Furthermore, it is statistically significant for richer individuals only and larger in size for both rich and highly educated ones. So the effect may be mediated by materialistic concerns but not so much by economic distress after all! The simplest explanation is that in our data rich and highly educated individuals have more environmentally-friendly political opinions in the first place and so there is more scope for them to shy away from these opinions. Another one is that uncertainty in energy prices had more severe consequences for entrepreneurs and richer individuals than for low-income workers, who were also subsidized by government across Europe in their energy consumption. Unfortunately, we have no way of testing for these explanations and we leave it for future work.

Finally, our results reveal an additional dimension of complexity. The war had relevant consequences on political opinions and concerns about climate change; yet, it did not affect people’s personal responsibility with environmental issues. This suggests that political preferences over the environment may be more sensitive to shifts in external conditions while basic pro-environmental attitudes may be more stable over time. This is reassuring for the future of the European Green Deal, as political shifts may not last forever and the underlying pro-environmental attitudes may move the pendulum back to a stronger support for eco-friendly policies.

## Supplementary Information

Below is the link to the electronic supplementary material.

Supplementary file 1 (pdf 296 KB)

## Footnotes

1 We document this increasing trend in pro-environmental attitudes in Appendix S4. It is outside of the scope of the paper to investigate its determinants, but it could be due to changes in the composition of the population over time or to the influence of the “Fridays for future” movements.

2 We downloaded this data from the ENTSO-E transparency portal. The data are originally hourly; we consider the monthly average in our analysis. For countries that have more than one bidding zone, e.g., Italy, we build a country-level average. These are the prices generators receive for selling electricity on the spot market. Fluctuations in these prices are translated to consumer prices usually after 1 or 2 months, while–to our advantage–not being differentiated by the type of consumer, e.g., businesses or consumers, or being affected by subsidies or taxes.

3 In Appendix S5 we consider different time lags for robustness, more specifically no lags, two and three months lags, and the results are qualitatively the same.

4 We miss data on personal responsibility and climate change concerns for 2018 and 2019. We substitute in these regressions the control for seasonality - because it produces collinearity with the time variables of interest - with a year trend. Anyway, as we show in Table S5 - 3 the exclusion of this control has no impact on our results.

5 Political orientation is identified using the ESS item on self-placement on a 0/10 left-right scale, where answers are coded for our analysis into three categories: 0–3 for left-wing, 4–6 for center, and 7–10 for right-wing political orientation. Interest into politics is an ESS item that asks to specify how much one is interested into politics in a 4-point Likert scale between “very” and “not interested”. We consider each possible answer as a separate category.
