# Supplementary material for: The impact of Ukraine’s war outbreak on green preferences in Europe
Source: Ambio. 2025 Apr 22;54(6):1095–102. doi: 10.1007/s13280-025-02173-1 (PMC12055698; doi:10.1007/s13280-025-02173-1)
Supplement: Supplementary file 1 — (pdf 296 KB) [file 13280_2025_2173_MOESM1_ESM.pdf]

Ambio

Supplementary Information

Title: **The impact of Ukraine's war outbreak on green preferences in Europe**

Appendix S1 Descriptive statistics

The war period (+1) covers from February 24, 2022, to May 23, 2022, while sub-period -1 ranges from August 24, 2021, to November 23, 2021, and sub-period -2 from May 24, 2021, to August 23, 2021. Observations outside this interval, accounting for just under 7%, were excluded due to the limited number of observations[6]. In some cases the interview can last more than one day and we have information on both the dates when the interviews starts and when they finish. If the start and end dates of an interview fall into different sub-periods, since it's unclear when responses to specific questions were given, that data was excluded. This involves a sample reduction of less than 1%.

Table S1 - 1: Descriptive stat. of the 5 dependent variables

	**Obs**	**Mean**	**Std. dev.**	**Min**	**Max**
Env. Salience	5408	5.3277	2.0394	1	10
Env. Position	5408	4.9175	2.1985	1	9
CL. Ch. Worry	5408	3.4345	0.9154	1	5
Per. Responsibility	5408	6.8597	2.2289	0	10
Env. Sensitivity	4484	4.9173	0.9785	1	6

Table S1 - 2: Descriptive stat. of the independent variables

	**Obs**	**Mean**	**Std. dev.**	**Min**	**Max**
Post	5408	0.3127	0.4636	0	1
Big City	5408	0.3291	0.4699	0	1
Female	5408	0.4702	0.4992	0	1
Income Perceived	5408	-	-	1	4
Education	5408	-	-	1	7
Age	5408	-	-	1	8
Activity	5408	-	-	1	9
Country	5408	-	-	1	9
Energy price (log - lag1)	5408	5.0705	0.5089	3.7015	6.1051
Google Trends	5408	13.2229	23.1754	0.5	100

Table S1 - 3: Descriptive statistics of Income perceived

	Freq.	Percent
Very difficult	170	3.14
Difficult	699	12.93
Coping	1951	36.08
Living comfortably	2588	47.86
Total	5408	100.00

Table S1 - 4: Descriptive statistics of Education (ISCED)

	Freq.	Percent
Less than lower secondary	379	7.01
Lower secondary	738	13.65

[6] All the results are robust even when all information are considered, i.e. when we include one quarter before 24rd May 2021 and two quarters after 23th May 2022. In this case the correspondent coefficients of these further periods are not significant with very large confidence intervals as a result of the low number of observations.

Lower tier upper secondary	632	11.69
Upper tier upper secondary	930	17.20
Advanced vocational	582	10.76
Lower tertiary education	1007	18.62
Higher tertiary education	1140	21.08
Total	5408	100.00

Table S1 - 5: Descriptive statistics of Age

	Freq.	Percent
15-20	214	3.96
21-30	565	10.45
31-40	701	12.96
41-50	886	16.38
51-60	1073	19.84
61-70	993	18.36
71-80	745	13.78
81-90	231	4.27
Total	5408	100.00

Table S1 - 6: Descriptive statistics of Activity

	Freq.	Percent
Paid work	2892	53.48
Education	342	6.32
Unemp. looking for job	137	2.53
Unemp. not looking for job	63	1.16
Permanent sick or disabled	147	2.72
Retired	1424	26.33
Housework	375	6.93
Total	28	0.52
Total	5408	100.00

Table S1 - 7: Descriptive statistics of Country

	Freq.	Percent
Belgium	564	10.43
Spain	753	13.92
the UK	517	9.56
Greece	740	13.68
Italy	512	9.47
Norway	881	16.29
Poland	161	2.98
Switzerland	642	11.87
the Netherlands	638	11.80
Total	5408	100.00

Appendix S2 Full models

This appendix presents the complete models of the results shown in the main body of the paper in Figure 2 and Table 1, to which it also adds models for the variable Sensitivity, which is not part of the main analysis as it is not available for some of the countries under investigation (Spain and Poland).

Table S2 - 1: Event study models

	**Env. Salience**	**Worry**	**Responsibility**	**Env. Position**	**Sensitivity**
Pre-Post Quorters					
+1 (24Feb-23May)	-0.0913**	-0.1082*	-0.0292	-0.1020***	-0.0352
-2 (24Aug-23Nov)	-0.0311	-0.0636	0.0161	-0.0164	0.0345
-3 (24May-23Aug)	0.0803	0.0216	0.0812	0.0582	0.0832
Quorters (ESS9-ESS8)	0.1536	0.0399	0.0601	-0.2673	0.1145
Big City	0.1366***	0.032	-0.0346	0.1794***	0.0517
Female	0.1211***	0.1915***	0.2296***	0.1780***	0.1251***
Income Perceived					
Difficult	0.0547	0.0329	-0.1978*	0.0795	-0.0906
Coping	-0.0319	-0.0657	-0.2580**	-0.0601	-0.1463
Living comfortably	-0.0272	-0.0985	-0.2665**	-0.0765	-0.1449
Education					
Less than lower sec.	-0.0236	-0.0093	-0.0796	-0.0329	-0.2412**
Lower secondary	-0.1214**	-0.051	-0.0709	-0.1110*	-0.0411
Lower upper sec.	-0.2319***	-0.0482	-0.0122	-0.1367**	-0.0058
Adv. Vocational	-0.0931	-0.0511	0.0537	-0.0326	-0.0278
Lower tertiary	0.0962*	0.1813***	0.1764***	0.1214**	0.1530**
Higher tertiary	0.2405***	0.3133***	0.1981***	0.3105***	0.2332***
Age					
15-20	0.0586	0.0799	-0.0849	0.0923	-0.0814
31-40	0.036	0.0451	0.0263	0.0814	-0.0781
41-50	-0.1076	0.036	0.1392*	-0.0038	0.0179
51-60	-0.0786	0.0351	0.2401***	0.0141	-0.0007
61-70	-0.1364*	0.1826**	0.2685***	-0.008	0.0713
71-80	-0.2379***	0.0181	0.2252**	-0.2016**	0.0944
81-90	-0.3712***	-0.1779	-0.0984	-0.3007***	-0.0487
Activity					
Education	0.1281	0.11	0.0713	0.2281**	0.0102
Un. looking for job	0.2155**	0.0747	-0.035	0.2545***	0.1799*
Un. not looking for job	0.0613	0.0105	-0.1631	-0.0378	0.0548
Permanent sick or dis.	-0.1481	-0.0374	0.0307	0.0404	0.1195
Retired	0.0497	0.0291	-0.1332**	0.1147*	0.0553
Military service	-0.4949	0.5860**	1.0368*	0.289	0.9909*
Housework	0.0825	0.0675	0.0249	0.0664	0.0843
Other	-0.2511	-0.089	-0.0709	-0.379	
Country					
Belgium	-0.0309	0.0779	0.0494	-0.0291	-0.0317
Spain	0.3806***	0.1837***	0.3825***	0.1059	
the UK	0.4376***	0.1009	0.4887***	0.6739***	-0.0564
Greece	-0.1987***	-0.1055	-0.2923***	-0.4265***	-0.4027***
Norway	0.3144***	-0.2509***	0.1773**	0.1102	-0.6948***
Poland	-0.1851***	-0.1982**	0.2275**	-0.6063***	
Switzerland	0.4192***	0.0114	0.4497***	0.2460***	-0.1636**
the Netherlands	0.4034***	-0.0542	0.2097***	-0.0656	-0.2887***

_cons	-0.2175*	-0.1192	-0.2106	-0.1799	0.2374
N	5026	5026	5026	5026	4116
adj. R-sq	0.1062	0.0481	0.0984	0.1314	0.0687
AIC	1.35E+04	1.41E+04	1.39E+04	1.35E+04	1.15E+04
BIC	1.37E+04	1.43E+04	1.42E+04	1.38E+04	1.17E+04

*** p<0.01, ** p<0.05, * p<0.10

Category reference classes: Income Perceived -Very Difficult-, Education -Upper secondary-, Age -21-30-, Activity -Paid Work-, Country -Italy- and Pre-Post Quorters - -1 (24Nov-23Feb)-

Table S2 - 2: Benchmark models

	**Env. Salience**	**Worry**	**Responsibility**	**Env. Position**	**Sensitivity**
Post	-0.0993***	-0.0884***	-0.0401	-0.0930***	-0.053
Big City	0.1539***	0.0846**	0.0094	0.1585***	0.0586
Female	0.1844***	0.2003***	0.2105***	0.2265***	0.1322***
Income Perceived					
Difficult	0.1341	0.0302	-0.2207**	0.1438*	-0.1686
Coping	0.1003	-0.098	-0.2854***	0.0494	-0.2164**
Living comfortably	0.1442*	-0.0859	-0.2631***	0.0454	-0.1677
Education					
Less than lower sec.	0.043	-0.0084	-0.0415	0.0167	-0.1954**
Lower secondary	-0.1226**	-0.0402	-0.1319**	-0.1492**	-0.0356
Lower upper sec.	-0.2262***	-0.0376	-0.0297	-0.1672**	0.0148
Adv. Vocational	-0.0519	-0.0028	0.0319	-0.0496	0.0702
Lower tertiary	0.1182**	0.2588***	0.1775***	0.1159**	0.1445**
Higher tertiary	0.2701***	0.3414***	0.1589***	0.2896***	0.2384***
Age					
15-20	0.0585	0.1002	-0.0799	0.0962	-0.1417
31-40	0.0597	0.0137	0.0797	0.1036	-0.0027
41-50	-0.0241	0.0197	0.1277*	0.0268	0.1123
51-60	-0.0118	0.0584	0.2204***	0.0363	0.0665
61-70	-0.1485*	0.1541*	0.2134***	-0.0577	0.1352
71-80	-0.1862**	0.0103	0.1035	-0.1935**	0.1106
81-90	-0.2235*	-0.0259	-0.1577	-0.2645**	0.0259
Activity					
Education	0.1752	0.1009	0.0153	0.2129**	0.0771
Un. looking for job	0.2631***	0.0825	-0.0548	0.2240*	0.2112**
Un. not looking for job	0.0205	0.2820*	0.0844	0.0754	0.085
Permanent sick or dis.	-0.0441	0.0471	0.0586	0.1303	0.1153
Retired	0.0663	-0.0527	-0.0856	0.1174**	0.1183
Housework	0.0625	0.0121	-0.0094	0.0556	0.0687
Other	-0.2723	0.099	0.0199	-0.3496*	
Country					
the UK	0.3747***	0.0467	0.4384***	0.6512***	-0.1046
Belgium	0.0242	0.0181	0.0553	0.1148	-0.1087*
Spain	0.3621***	0.1680***	0.3658***	0.1077*	
Norway	0.2895***	-0.2989***	0.1736***	0.1221*	-0.6838***
Poland	-0.1789**	-0.1062	0.2572**	-0.6137***	
Switzerland	0.3857***	-0.0152	0.4408***	0.2467***	-0.1232
the Netherlands	0.3687***	-0.0667	0.1935***	-0.038	-0.3240***
Greece	-0.1300*	-0.1700***	-0.3717***	-0.3354***	-0.4351***
_cons	-0.4483***	-0.1409	-0.1354	-0.3338***	0.2119
N	5408	5408	5408	5408	4484

adj. R-sq	0.0953	0.0605	0.1001	0.124	0.0695
AIC	1.48E+04	1.50E+04	1.47E+04	1.46E+04	1.26E+04
BIC	1.50E+04	1.52E+04	1.49E+04	1.49E+04	1.28E+04

*** p<0.01, ** p<0.05, * p<0.10

Category reference classes: Income Perceived -Very difficult-, Education -Upper secondary-, Age -21-30-, Activity -Paid Work- and Country -Italy-

Table S2 - 3: Benchmark models with Google Trends

	**Env. Salience**	**Worry**	**Responsibility**	**Env. Position**	**Sensitivity**
Post	-0.0761*	-0.0683*	-0.015	-0.0775*	-0.0549
Google Trends	-0.0008	-0.0007	-0.0009	-0.0005	0.0001
Big City	0.1552***	0.0857**	0.0108	0.1593***	0.0584
Female	0.1829***	0.1990***	0.2089***	0.2255***	0.1323***
Income Perceived					
Difficult	0.1318	0.0283	-0.2232**	0.1422*	-0.1685
Coping	0.0999	-0.0983	-0.2859***	0.0491	-0.2165**
Living comfortably	0.1438*	-0.0862	-0.2635***	0.0452	-0.1677
Education					
Less than lower sec.	0.0446	-0.007	-0.0397	0.0178	-0.1955**
Lower secondary	-0.1233**	-0.0408	-0.1327**	-0.1496**	-0.0355
Lower upper sec.	-0.2248***	-0.0364	-0.0282	-0.1663**	0.0147
Adv. Vocational	-0.0525	-0.0033	0.0312	-0.05	0.0703
Lower tertiary	0.1195**	0.2599***	0.1789***	0.1167**	0.1445**
Higher tertiary	0.2706***	0.3418***	0.1594***	0.2900***	0.2383***
Age					
15-20	0.0572	0.099	-0.0813	0.0953	-0.1416
31-40	0.0589	0.013	0.0788	0.103	-0.0027
41-50	-0.0248	0.0192	0.1270*	0.0264	0.1123
51-60	-0.0121	0.0582	0.2201***	0.0361	0.0665
61-70	-0.1485*	0.1541*	0.2134***	-0.0577	0.1352
71-80	-0.1860**	0.0105	0.1036	-0.1934**	0.1106
81-90	-0.2243*	-0.0266	-0.1586	-0.2650**	0.0259
Activity					
Education	0.1774	0.1027	0.0176	0.2143**	0.0769
Un. looking for job	0.2599***	0.0797	-0.0583	0.2219*	0.2114**
Un. not looking for job	0.0211	0.2825*	0.0851	0.0758	0.085
Permanent sick or dis.	-0.0426	0.0484	0.0602	0.1313	0.1151
Retired	0.0663	-0.0527	-0.0856	0.1174**	0.1182
Housework	0.0651	0.0143	-0.0066	0.0573	0.0684
Other	-0.2764	0.0955	0.0154	-0.3524*	
Country					
Belgium	0.0163	0.0113	0.0468	0.1095	-0.1080*
Spain	0.3685***	0.1735***	0.3727***	0.1120*	
the UK	0.3673***	0.0403	0.4303***	0.6462***	-0.104
Greece	-0.1279*	-0.1682***	-0.3695***	-0.3340***	-0.4353***
Norway	0.2868***	-0.3013***	0.1707***	0.1202*	-0.6836***
Poland	-0.1659**	-0.095	0.2713**	-0.6050***	
Switzerland	0.3881***	-0.0131	0.4434***	0.2483***	-0.1234
the Netherlands	0.3727***	-0.0632	0.1979***	-0.0353	-0.3243***
_cons	-0.4437***	-0.1368	-0.1304	-0.3307***	0.2116
N	5408	5408	5408	5408	4484
adj. R-sq	0.0954	0.0605	0.1003	0.1239	0.0693

AIC	1.48E+04	1.50E+04	1.47E+04	1.46E+04	1.26E+04
BIC	1.50E+04	1.52E+04	1.49E+04	1.49E+04	1.28E+04

*** p<0.01, ** p<0.05, * p<0.10

Category reference classes: Income Perceived -Very Difficult-, Education -Upper secondary-, Age -21-30-, Activity -Paid Work- and Country -Italy-

Table S2 - 4: Benchmark models with Energy prices

	**Env. Salience**	**Worry**	**Responsibility**	**Env. Position**	**Sensitivity**
Post	-0.0715**	-0.0829**	-0.0172	-0.0692**	-0.0467
Energy price (log - lag1)	-0.1434**	-0.0286	-0.1178**	-0.1227*	-0.0266
Big City	0.1527***	0.0843**	0.0084	0.1574***	0.0583
Female	0.1847***	0.2004***	0.2107***	0.2267***	0.1324***
Income Perceived					
Difficult	0.1392*	0.0313	-0.2166**	0.1481*	-0.1675
Coping	0.1038	-0.0973	-0.2826***	0.0524	-0.2157**
Living comfortably	0.1477*	-0.0852	-0.2603***	0.0484	-0.1669
Education					
Less than lower sec.	0.0419	-0.0086	-0.0423	0.0158	-0.1955**
Lower secondary	-0.1243**	-0.0405	-0.1333**	-0.1506**	-0.0358
Lower upper sec.	-0.2290***	-0.0381	-0.032	-0.1696**	0.0141
Adv. Vocational	-0.0528	-0.003	0.0311	-0.0503	0.07
Lower tertiary	0.1158**	0.2583***	0.1755***	0.1138**	0.1440**
Higher tertiary	0.2682***	0.3410***	0.1573***	0.2880***	0.2378***
Age					
15-20	0.0616	0.1008	-0.0774	0.0989	-0.1418
31-40	0.0605	0.0138	0.0804	0.1043	-0.0029
41-50	-0.0238	0.0198	0.1279*	0.0271	0.1122
51-60	-0.0113	0.0585	0.2208***	0.0367	0.0662
61-70	-0.1508**	0.1536*	0.2114***	-0.0598	0.1342
71-80	-0.1880**	0.01	0.102	-0.1951**	0.1098
81-90	-0.2234*	-0.0259	-0.1576	-0.2644**	0.0254
Activity					
Education	0.1698	0.0998	0.0108	0.2082**	0.0758
Un. looking for job	0.2568**	0.0813	-0.06	0.2186*	0.2098**
Un. not looking for job	0.0166	0.2812*	0.0812	0.0721	0.0838
Permanent sick or dis.	-0.048	0.0463	0.0554	0.127	0.1144
Retired	0.0636	-0.0532	-0.0878	0.1150**	0.1176
Housework	0.0603	0.0117	-0.0112	0.0537	0.068
Other	-0.2678	0.0999	0.0236	-0.3458*	
Country					
Belgium	-0.0101	0.0113	0.0271	0.0854	-0.1151*
Spain	0.3379***	0.1632***	0.3459***	0.087	
the UK	0.3514***	0.0421	0.4192***	0.6313***	-0.1089
Greece	-0.1423**	-0.1724***	-0.3819***	-0.3460***	-0.4375***
Norway	0.1247	-0.3318***	0.0382	-0.019	-0.7144***
Poland	-0.2652***	-0.1235	0.1863	-0.6876***	
Switzerland	0.3252***	-0.0273	0.3911***	0.1949*	-0.1344
the Netherlands	0.3174***	-0.0769	0.1514**	-0.0819	-0.3335***
_cons	0.3263	0.0137	0.501	0.329	0.3556
N	5408	5408	5408	5408	4484
adj. R-sq	0.0964	0.0604	0.1008	0.1247	0.0693
AIC	1.48E+04	1.50E+04	1.47E+04	1.46E+04	1.26E+04

BIC	1.50E+04	1.52E+04	1.49E+04	1.49E+04	1.28E+04

*** p<0.01, ** p<0.05, * p<0.10

Category reference classes: Income Perceived -Very Difficult-, Education -Upper secondary-, Age -21-30-, Activity -Paid Work- and Country -Italy-

Table S2 - 5: Models with only Energy prices

	**Env. Salience**	**Worry**	**Responsibility**	**Env. Position**	**Sensitivity**
Energy price (log - lag1)	-0.1922***	-0.0851	-0.1295**	-0.1699**	-0.0595
Big City	0.1519***	0.0834**	0.0082	0.1566***	0.0577
Female	0.1849***	0.2007***	0.2108***	0.2269***	0.1330***
Income Perceived	0	0	0	0	0
Difficult	0.1409*	0.0332	-0.2162**	0.1498*	-0.166
Coping	0.1053	-0.0955	-0.2822***	0.0538	-0.2147**
Living comfortably	0.1495*	-0.0831	-0.2598***	0.0501	-0.1654
Education					
Less than lower sec.	0.0415	-0.009	-0.0424	0.0155	-0.1953**
Lower secondary	-0.1253**	-0.0418	-0.1335**	-0.1516**	-0.0365
Lower upper sec.	-0.2306***	-0.04	-0.0324	-0.1711**	0.0127
Adv. Vocational	-0.0527	-0.0029	0.0311	-0.0503	0.0696
Lower tertiary	0.1150**	0.2574***	0.1754***	0.1131**	0.1431**
Higher tertiary	0.2671***	0.3397***	0.1571***	0.2869***	0.2358***
Age					
15-20	0.064	0.1036	-0.0768	0.1012	-0.1425
31-40	0.0612	0.0146	0.0805	0.1049	-0.0037
41-50	-0.0234	0.0203	0.1280*	0.0275	0.1117
51-60	-0.0109	0.0591	0.2209***	0.0372	0.0648
61-70	-0.1518**	0.1525*	0.2112***	-0.0607	0.1313
71-80	-0.1888**	0.0091	0.1018	-0.1959**	0.1077
81-90	-0.2258*	-0.0288	-0.1582	-0.2668**	0.0218
Activity					
Education	0.1692	0.099	0.0107	0.2076**	0.0743
Un. looking for job	0.2541**	0.0782	-0.0606	0.2160*	0.2100**
Un. not looking for job	0.0154	0.2797*	0.0809	0.0709	0.0795
Permanent sick or dis.	-0.0477	0.0466	0.0555	0.1272	0.1135
Retired	0.0643	-0.0524	-0.0877	0.1157**	0.1172
Housework	0.0604	0.0117	-0.0112	0.0538	0.0672
Other	-0.2657	0.1024	0.0241	-0.3438*	
Country					
Belgium	-0.0219	-0.0023	0.0243	0.074	-0.1230**
Spain	0.3297***	0.1537***	0.3439***	0.079	
the UK	0.3433***	0.0327	0.4173***	0.6235***	-0.1142*
Greece	-0.1465**	-0.1772***	-0.3829***	-0.3500***	-0.4405***
Norway	0.0684	-0.3971***	0.0246	-0.0734	-0.7523***
Poland	-0.2928***	-0.1555	0.1797	-0.7143***	
Switzerland	0.3047***	-0.051	0.3861***	0.1751	-0.1481
the Netherlands	0.2998***	-0.0973	0.1472**	-0.0989	-0.3453***
_cons	0.5582	0.2824	0.5569*	0.5534	0.5143
N	5408	5408	5408	5408	4484
adj. R-sq	0.0954	0.059	0.1009	0.1238	0.0691
AIC	1.48E+04	1.50E+04	1.47E+04	1.46E+04	1.26E+04
BIC	1.50E+04	1.52E+04	1.49E+04	1.49E+04	1.28E+04

*** p<0.01, ** p<0.05, * p<0.10

Category reference classes: Income Perceived -Very Difficult-, Education -Upper secondary-, Age -21-30-, Activity -Paid Work- and Country -Italy-

Table S2 - 6: Models with only Google Trends

	Env. Salience	Worry	Responsibility	Env. Position	Sensitivity
Google Trends	-0.0016**	-0.0014**	-0.001	-0.0013*	-0.0005
Big City	0.1561***	0.0865**	0.011	0.1602***	0.0593
Female	0.1815***	0.1978***	0.2086***	0.2241***	0.1316***
Income Perceived					
Difficult	0.1295	0.0262	-0.2237**	0.1399*	-0.1694
Coping	0.0998	-0.0984	-0.2859***	0.049	-0.2160**
Living comfortably	0.1440*	-0.0861	-0.2635***	0.0453	-0.1667
Education					
Less than lower sec.	0.0462	-0.0055	-0.0394	0.0195	-0.1945**
Lower secondary	-0.1244**	-0.0418	-0.1329**	-0.1508**	-0.0366
Lower upper sec.	-0.2240***	-0.0357	-0.0281	-0.1655**	0.0154
Adv. Vocational	-0.0528	-0.0036	0.0312	-0.0503	0.0694
Lower tertiary	0.1208**	0.2611***	0.1792***	0.1181**	0.1443**
Higher tertiary	0.2707***	0.3419***	0.1595***	0.2900***	0.2379***
Age					
15-20	0.057	0.0988	-0.0814	0.0952	-0.1435
31-40	0.0585	0.0126	0.0787	0.1026	-0.0031
41-50	-0.0251	0.0189	0.1269*	0.0261	0.1121
51-60	-0.0121	0.0581	0.2201***	0.0361	0.0656
61-70	-0.1487*	0.1539*	0.2133***	-0.0579	0.1336
71-80	-0.1860**	0.0105	0.1037	-0.1934**	0.1096
81-90	-0.2273*	-0.0293	-0.1592	-0.2681**	0.0228
Activity					
Education	0.1805	0.1055	0.0182	0.2174**	0.0792
Un. looking for job	0.2562***	0.0764	-0.059	0.2181*	0.2116**
Un. not looking for job	0.0218	0.2831*	0.0852	0.0765	0.0831
Permanent sick or dis.	-0.0397	0.0509	0.0608	0.1342	0.1174
Retired	0.0676	-0.0515	-0.0854	0.1187**	0.1192
Housework	0.0683	0.0172	-0.006	0.0606	0.0703
Other	-0.28	0.0922	0.0147	-0.3561*	
Country					
Belgium	0.0084	0.0042	0.0452	0.1014	-0.1141*
Spain	0.3748***	0.1792***	0.3739***	0.1184*	
the UK	0.3597***	0.0336	0.4289***	0.6386***	-0.1096
Greece	-0.1259*	-0.1663***	-0.3691***	-0.3319***	-0.4336***
Norway	0.2839***	-0.3039***	0.1701***	0.1173*	-0.6858***
Poland	-0.1514**	-0.082	0.2742**	-0.5903***	
Switzerland	0.3906***	-0.0109	0.4439***	0.2508***	-0.1215
the Netherlands	0.3766***	-0.0597	0.1987***	-0.0313	-0.3213***
_cons	-0.4661***	-0.157	-0.1348	-0.3535***	0.1963
N	5408	5408	5408	5408	4484
adj. R-sq	0.0946	0.0599	0.1005	0.123	0.069
AIC	1.48E+04	1.50E+04	1.47E+04	1.46E+04	1.26E+04
BIC	1.50E+04	1.52E+04	1.49E+04	1.49E+04	1.28E+04

*** p<0.01, ** p<0.05, * p<0.10

Category reference classes: Income Perceived -Very Difficult-, Education -Upper secondary-, Age -21-30-, Activity -Paid Work- and Country -Italy-

Table S2 - 7: Models for seasonal trends

	**Env. Salience**	**Worry**	**Responsibility**	**Env. Position**	**Sensitivity**
Month					
February	0.0515	-0.0046	0.009	0.058	0.0191
March	0.0011	0.2115	0.12	0.0057	-0.0954*
April	0.0638	0.0575	0.106	0.0356	0.1107
May	0.0593	0.0845	0.2232	0.0511	0.1408
June	0.248	0.2835	-0.3597	0.0512	0.6979***
July	0	0	0	0	0
August	0.0775	0.0904	0.016	0.0289	-0.1933
September	0.1786***	-0.0231	-0.028	0.1263***	-0.0640*
October	0.1322***	-0.0145	0.0085	0.0827**	-0.0649*
November	0.1249***	-0.0584	-0.0685	0.0639*	-0.0268
December	0.0588	0.0066	-0.0543	-0.0101	-0.0174
Big City	0.1391***	0.1171***	0.0591**	0.1359***	-0.0276
Female	0.1409***	0.1127***	0.0742***	0.1551***	0.0883***
Income Perceived					
Difficult	0.1088*	-0.1487	0.0547	0.0313	-0.1126*
Coping	0.0448	-0.2985***	0.0138	-0.0312	-0.1698***
Living comfortably	-0.0378	-0.3643***	0.0289	-0.1627***	-0.1747***
Education					
Less than lower sec.	-0.1390***	-0.2088***	-0.2373***	-0.1448***	-0.1405***
Lower secondary	-0.1558***	-0.1444***	-0.1502***	-0.1443***	-0.1227***
Lower upper sec.	-0.2035***	-0.1436***	-0.0842*	-0.1540***	-0.0689*
Adv. Vocational	-0.0382	-0.0831*	0.022	-0.0498	0.0362
Lower tertiary	0.1383***	0.0940**	0.1660***	0.1325***	0.0879***
Higher tertiary	0.2383***	0.1135**	0.1974***	0.2129***	0.1432***
Age					
15-20	-0.0447	0.0676	0.0559	-0.069	-0.0861
31-40	-0.0346	0.0241	0.1854***	-0.0207	0.1316***
41-50	-0.1010***	-0.0498	0.1497***	-0.0804**	0.1364***
51-60	-0.0368	-0.0717	0.1397***	-0.0297	0.2774***
61-70	-0.1042**	-0.0264	0.1668***	-0.0874**	0.3030***
71-80	-0.1966***	-0.1085	0.1076	-0.1921***	0.2686***
81-90	-0.2198***	-0.2599***	-0.3872***	-0.1903***	0.3140***
Activity					
Education	0.2029***	0.2169***	0.1699**	0.2285***	0.1790***
Un. looking for job	0.1590***	-0.0241	-0.0114	0.1598***	0.1191**
Un. not looking for job	0.2021**	0.0238	0.0067	0.119	-0.0559
Permanent sick or dis.	0.0624	0.1382*	0.1224	0.0494	0.0876
Retired	-0.0075	-0.0596	-0.1165**	-0.0023	0.0880***
Military service	-0.1008	0.1118	0.2057	-0.0456	0.0812
Housework	-0.0634	-0.0071	-0.0093	-0.0585	0.0994***
Other	0.0237			0.0312	0.3163***
Country					
Spain	0.2149***	0.102	0.0239	-0.1263***	0.1021***
the UK	-0.1147***	-0.3226***	0.0914*	0.1142***	-0.1446***
Belgium	-0.2399***	-0.0071	0.1325**	-0.2131***	-0.0553
Norway	0.0147	-0.3537***	0.1198**	-0.2418***	-0.5131***
Poland	-0.4942***	-0.4993***	0.0211	-1.1320***	-0.0107
Switzerland	0.0850*	-0.1139**	0.4535***	-0.0814*	0.1426***

the Netherlands	0.0447	-0.1686***	0.054	-0.3951***	-0.1655***
_cons	-0.1003	0.4744***	-0.2528*	0.1793**	0.0309
N	12614	6221	6221	12614	12534
adj. R-sq	0.0729	0.0841	0.0698	0.1303	0.0613
AIC	3.49E+04	1.71E+04	1.72E+04	3.41E+04	3.47E+04
BIC	3.52E+04	1.74E+04	1.75E+04	3.44E+04	3.51E+04

*** p<0.01, ** p<0.05, * p<0.10

Category reference classes: Month -January-, Income Perceived -Very Difficult-, Education -Upper secondary-, Age -21-30-, Activity -Paid Work-, Country -Italy- and Pre-Post Quorters - -1 (24Nov-23Feb)-

Table S2 - 8: Placebo event study on 2016-2019

	**Env. Salience**	**Worry**	**Responsibility**	**Env. Position**	**Sensitivity**
Quarters					
24May-23Aug	0.0126	0.2062	-0.0298	-0.1301	0.039
24Aug-23Nov	0.1052***	-0.0378	0.0239	0.0763***	0.0006
24Feb-23May	-0.0186	0.2405***	0.1415*	0.004	-0.0219
Big City	0.1402***	0.1184***	0.0577**	0.1374***	-0.0262
Female	0.1410***	0.1136***	0.0759***	0.1554***	0.0895***
Income Perceived					
Difficult	0.1094*	-0.1435	0.0567	0.031	-0.1162*
Coping	0.0468	-0.2930***	0.0165	-0.0303	-0.1671***
Living comfortably	-0.0361	-0.3579***	0.0325	-0.1619***	-0.1701***
Education					
Less than lower sec.	-0.1347***	-0.2027***	-0.2374***	-0.1416***	-0.1335***
Lower secondary	-0.1546***	-0.1400***	-0.1495***	-0.1425***	-0.1173***
Lower upper sec.	-0.2019***	-0.1355***	-0.0763	-0.1514***	-0.0650*
Adv. Vocational	-0.0382	-0.0769	0.0236	-0.0492	0.0372
Lower tertiary	0.1390***	0.0976**	0.1668***	0.1335***	0.0888***
Higher tertiary	0.2384***	0.1211***	0.2002***	0.2124***	0.1436***
Age					
15-20	-0.0434	0.0718	0.0579	-0.0681	-0.088
31-40	-0.0339	0.0267	0.1906***	-0.0179	0.1335***
41-50	-0.1003***	-0.0457	0.1551***	-0.0779**	0.1400***
51-60	-0.0371	-0.0662	0.1437***	-0.029	0.2800***
61-70	-0.1034**	-0.0196	0.1725***	-0.0840**	0.3042***
71-80	-0.1992***	-0.1019	0.1109	-0.1895***	0.2617***
81-90	-0.2223***	-0.2478***	-0.3718***	-0.1903***	0.3059***
Activity					
Education	0.1987***	0.2247***	0.1786***	0.2277***	0.1738***
Un. looking for job	0.1652***	-0.0285	-0.0101	0.1659***	0.1258**
Un. not looking for job	0.1842**	0.0197	0.0076	0.1092	-0.0724
Permanent sick or dis.	0.0648	0.1391*	0.1217	0.0513	0.0886
Retired	-0.0045	-0.0639	-0.1183**	-0.0007	0.0896***
Military	-0.12	0.1098	0.1979	-0.0701	0.0289
Housework	-0.0621	-0.0091	-0.0122	-0.0563	0.1000***
Other	-0.0037			0.0156	0.2538**
Year	0.0300***	-0.0127	0.0437	0.0171**	0.0630***
Country					
Spain	0.2454***	0.002	0.0344	-0.1182***	0.1415***
the UK	-0.0756**	-0.3414***	0.1356*	0.1309***	-0.1085***
Belgium	-0.1984***	-0.0251	0.1752**	-0.2016***	-0.0095
Norway	0.0517	-0.3648***	0.1631*	-0.2310***	-0.4664***

Poland	-0.4595***	-0.5352***	0.0436	-1.1343***	0.0474
Switzerland	0.1378***	-0.1335	0.4912***	-0.058	0.1809***
the Netherlands	0.0853**	-0.1886**	0.0933	-0.3839***	-0.1216***
_cons	-0.1675**	0.4991***	-0.3927**	0.1381*	-0.1973**
N	12640	6231	6231	12640	12560
adj. R-sq	0.073	0.085	0.0699	0.1304	0.0645
AIC	3.50E+04	1.71E+04	1.72E+04	3.42E+04	3.47E+04
BIC	3.52E+04	1.74E+04	1.75E+04	3.44E+04	3.50E+04

*** p<0.01, ** p<0.05, * p<0.10

Category reference classes: Income Perceived -Very Difficult-, Education -Upper secondary-, Age -21-30-, Activity -Paid Work-, Country -Italy- and Quorters -(24Nov-23Feb)-

Appendix S3 Heterogeneity

This appendix shows the complete models of the heterogeneity analysis of the results for subjects disaggregated by income level and education.

Table S3 - 1: Benchmark models with income interaction

	**Env. Salience**	**Worry**	**Responsibility**	**Env. Position**	**Sensitivity**
Post	-0.1129***	-0.0787**	-0.0384	-0.1046***	-0.0503
Poor	-0.1856**	0.1157	0.2681***	-0.081	0.1762
Post#Poor	0.082	-0.059	-0.01	0.0705	-0.0169
Big City	0.1536***	0.0848**	0.0095	0.1582***	0.0586
Female	0.1843***	0.2004***	0.2105***	0.2264***	0.1323***
Income Perceived	0	0	0	0	0
Difficult	0.134	0.0303	-0.2207**	0.1437*	-0.1686
Coping	-0.0441	-0.012	-0.0223	0.0038	-0.0487
Living comfortably	0	0	0	0	0
Education	0	0	0	0	0
Less than lower sec.	0.0458	-0.0104	-0.0418	0.0192	-0.1959**
Lower secondary	-0.1218**	-0.0408	-0.1320**	-0.1484**	-0.036
Lower upper sec.	-0.2256***	-0.038	-0.0298	-0.1667**	0.0147
Adv. Vocational	-0.0516	-0.003	0.0318	-0.0493	0.0702
Lower tertiary	0.1184**	0.2586***	0.1775***	0.1161**	0.1446**
Higher tertiary	0.2701***	0.3414***	0.1589***	0.2896***	0.2384***
Age	0	0	0	0	0
15-20	0.0568	0.1014	-0.0797	0.0947	-0.1411
31-40	0.0594	0.0139	0.0797	0.1033	-0.0027
41-50	-0.0248	0.0202	0.1277*	0.0263	0.1124
51-60	-0.0116	0.0583	0.2204***	0.0365	0.0664
61-70	-0.1477*	0.1536*	0.2133***	-0.0571	0.1351
71-80	-0.1863**	0.0104	0.1035	-0.1937**	0.1106
81-90	-0.2227*	-0.0265	-0.1578	-0.2638**	0.0258
Activity	0	0	0	0	0
Education	0.1749	0.1012	0.0153	0.2125**	0.0772
Un. looking for job	0.2605***	0.0844	-0.0545	0.2218*	0.2116**
Un. not looking for job	0.0225	0.2805*	0.0842	0.0771	0.0847
Permanent sick or dis.	-0.0455	0.0481	0.0588	0.1291	0.1156
Retired	0.0653	-0.0519	-0.0855	0.1165**	0.1185
Housework	0.0609	0.0133	-0.0092	0.0542	0.069
Other	-0.2757	0.1015	0.0203	-0.3526*	
Country	0	0	0	0	0

Belgium	0.0243	0.018	0.0553	0.1149	-0.1088*
Spain	0.3614***	0.1685***	0.3659***	0.1071	
the UK	0.3731***	0.0478	0.4386***	0.6499***	-0.1044
Greece	-0.1303*	-0.1697***	-0.3717***	-0.3357***	-0.4351***
Norway	0.2883***	-0.2981***	0.1738***	0.1210*	-0.6836***
Poland	-0.1811**	-0.1046	0.2575**	-0.6156***	
Switzerland	0.3851***	-0.0148	0.4409***	0.2462***	-0.1231
the Netherlands	0.3676***	-0.0659	0.1937***	-0.0389	-0.3238***
_cons	-0.2964***	-0.2324***	-0.3995***	-0.2817***	0.0427
N	5408	5408	5408	5408	4484
adj. R-sq	0.0953	0.0604	0.1	0.124	0.0693
AIC	1.48E+04	1.50E+04	1.47E+04	1.46E+04	1.26E+04
BIC	1.50E+04	1.52E+04	1.49E+04	1.49E+04	1.28E+04
Wald test P-value Post + Post#Low Educ.	0.6614	0.0762	0.5315	0.6069	0.4477

*** p<0.01, ** p<0.05, * p<0.10

Category reference classes: Income Perceived -Very Difficult-, Education -Upper secondary-, Age -21-30-, Activity -Paid Work- and Country -Italy-

Table S3 - 2: Benchmark models with education interaction

	**Env. Salience**	**Worry**	**Responsibility**	**Env. Position**	**Sensitivity**
Post	-0.1280**	-0.0787	-0.0783	-0.1155**	-0.1315**
Low Education	-0.2939***	-0.3333***	-0.1905***	-0.3083***	-0.3021***
Post#Low Educ.	0.0475	-0.0161	0.0633	0.0373	0.1276
Big City	0.1546***	0.0844**	0.0103	0.1590***	0.0603
Female	0.1839***	0.2005***	0.2098***	0.2261***	0.1304***
Income Perceived					
Difficult	0.1347	0.03	-0.2200**	0.1442*	-0.1682
Coping	0.1004	-0.098	-0.2853***	0.0495	-0.2184**
Living comfortably	0.1447*	-0.0861	-0.2624***	0.0458	-0.1675
Education					
Less than lower sec.	0.0435	-0.0086	-0.0407	0.0172	-0.1949**
Lower secondary	-0.1224**	-0.0403	-0.1316**	-0.1490**	-0.0354
Lower upper sec.	-0.2262***	-0.0376	-0.0297	-0.1672**	0.0147
Adv. Vocational	-0.0515	-0.0029	0.0324	-0.0493	0.0706
Lower tertiary	-0.1521***	-0.0825	0.0184	-0.1739***	-0.0948
Higher tertiary	0	0	0	0	0
Age					
15-20	0.0587	0.1001	-0.0796	0.0964	-0.1399
31-40	0.059	0.0139	0.0787	0.103	-0.0034
41-50	-0.0247	0.0199	0.1268*	0.0263	0.112
51-60	-0.0129	0.0588	0.2189***	0.0354	0.0638
61-70	-0.1508*	0.1549*	0.2103***	-0.0595	0.1292
71-80	-0.1886**	0.0112	0.1002	-0.1955**	0.1043
81-90	-0.2253*	-0.0253	-0.16	-0.2659**	0.0218
Activity					
Education	0.1745	0.1012	0.0143	0.2123**	0.073
Un. looking for job	0.2641***	0.0822	-0.0535	0.2248*	0.2107**
Un. not looking for job	0.0244	0.2807*	0.0897	0.0785	0.0983
Permanent sick or dis.	-0.0426	0.0466	0.0606	0.1315	0.1198
Retired	0.0674	-0.053	-0.0841	0.1182**	0.1216*
Housework	0.0624	0.0122	-0.0096	0.0555	0.0682

Other	-0.2728	0.0992	0.0192	-0.3501*	
Country					
Belgium	0.0246	0.018	0.0559	0.1151	-0.1079*
Spain	0.3612***	0.1683***	0.3646***	0.107	
the UK	0.3742***	0.0469	0.4377***	0.6508***	-0.106
Greece	-0.1300*	-0.1699***	-0.3718***	-0.3354***	-0.4355***
Norway	0.2884***	-0.2985***	0.1721***	0.1212*	-0.6871***
Poland	-0.1789**	-0.1062	0.2572**	-0.6137***	
Switzerland	0.3864***	-0.0154	0.4417***	0.2472***	-0.1217
the Netherlands	0.3682***	-0.0665	0.1928***	-0.0384	-0.3257***
_cons	-0.163	0.1953	0.0437	-0.0323	0.4934***
N	5408	5408	5408	5408	4484
adj. R-sq	0.0952	0.0603	0.1002	0.1239	0.0702
AIC	1.48E+04	1.50E+04	1.47E+04	1.46E+04	1.26E+04
BIC	1.50E+04	1.52E+04	1.49E+04	1.49E+04	1.28E+04
Wald test P-value Post + Post#Low Educ.	0.0553	0.0286	0.7196	0.0629	0.9407

*** p<0.01, ** p<0.05, * p<0.10
Category reference classes: Income Perceived -Very Difficult-, Education -Upper secondary-, Age -21-30-, Activity -Paid Work- and Country -Italy-

Appendix S4 Trends in enviromental preferences and voting

In this appendix, we test for the presence of a trend over our entire sample. In fact, the presence of a declining trend would pose a serious threat to our identification strategy. We deal with this in several ways. First, we include in the regressions controls for seasonality effects (see section 3 for details). If there was a declining trend over time, this should be captured by this control. Then, we directly tested for these trends over a time period ranging from 2016 to 2022, both excluding and including the COVID year 2020. The year 2020 is not considered in our main analyses so as not to fall into error from variation in people's preferences in the pandemic period. However, this group, interviewed at the tail end of wave 9, is very small. Moreover, it is important to note that the Cl. Ch. Worry and Personal Responsibility variables were not included in the COVID year 2020 test (Table S4 - 2), as they are not part of wave 9, and the results did not change compared to the models in Table S4 - 1. The results obtained reinforce those of our main analysis, as we find a significant and increasing trend.

Table S4 - 1: Models for daily trends before war - 2016-2019 and 2021-2022

	**Env. Salience**	**Worry**	**Responsibility**	**Env. Position**	**Sensitivity**
TREND	0.00005***	0.00015***	0.00017***	0.00007***	0.00003***
Big City	0.1443***	0.0792***	0.031	0.1424***	-0.0116
Female	0.1440***	0.1497***	0.1362***	0.1701***	0.1101***
Income Perceived					
Difficult	0.1028*	-0.0937	-0.0376	0.0281	-0.1468***
Coping	0.0416	-0.2477***	-0.0878	-0.0431	-0.2086***
Living comfortably	-0.0133	-0.2735***	-0.0699	-0.1453***	-0.1902***
Education					
Less than lower sec.	-0.0989***	-0.2106***	-0.2351***	-0.1204***	-0.1537***
Lower secondary	-0.1587***	-0.1307***	-0.1662***	-0.1545***	-0.1125***
Lower upper sec.	-0.2187***	-0.1329***	-0.1014***	-0.1624***	-0.0860***
Adv. Vocational	-0.0484	-0.0575	0.0116	-0.0567*	0.0276

Lower tertiary	0.1342***	0.1455***	0.1675***	0.1375***	0.0953***
Higher tertiary	0.2416***	0.1820***	0.1872***	0.2283***	0.1549***
Age					
15-20	-0.0283	0.1104*	0.0487	-0.0334	-0.0723
31-40	-0.0164	0.0465	0.1512***	-0.0111	0.1165***
41-50	-0.1148***	-0.0139	0.1491***	-0.0790**	0.1266***
51-60	-0.0624*	-0.029	0.1624***	-0.041	0.2373***
61-70	-0.1203***	0.0643	0.2000***	-0.0803**	0.2881***
71-80	-0.2145***	-0.0322	0.1084*	-0.1999***	0.2543***
81-90	-0.2649***	-0.1661**	-0.2853***	-0.2263***	0.2619***
Activity					
Education	0.2020***	0.1632***	0.1314**	0.2382***	0.1428***
Un. looking for job	0.1624***	-0.0129	-0.0358	0.1686***	0.0969**
Un. not looking for job	0.1447*	0.0625	-0.0105	0.0743	-0.0531
Permanent sick or dis.	0.0649	0.1388**	0.1200*	0.0668	0.0945*
Retired	0.0085	-0.0413	-0.1002***	0.024	0.0661**
Military	-0.0677	0.0979	0.2127	-0.0069	0.0999
Housework	-0.0314	0.0063	-0.0126	-0.0354	0.0869***
Other	-0.0897	-0.1864	-0.0775	-0.1018	0.3083***
Country					
Belgium	-0.1522***	0.0375	0.1728***	-0.1475***	-0.0785**
Spain	0.2162***	0.2357***	0.2079***	-0.1137***	0.1132***
the UK	0.0014	-0.2033***	0.1998***	0.2083***	-0.1670***
Greece	-0.4669***	-0.1735***	-0.3252***	-0.6902***	-0.4015***
Norway	0.0853***	-0.2694***	0.1736***	-0.1765***	-0.5331***
Poland	-0.4333***	-0.3736***	0.0667	-1.0754***	0.0032
Switzerland	0.2346***	-0.0201	0.4957***	0.0243	0.0464
the Netherlands	0.1489***	-0.1086**	0.1259***	-0.3281***	-0.1997***
_cons	-0.1398**	0.1035	-0.3992***	0.1248*	0.0429
N	16357	9948	9948	16357	15688
adj. R-sq	0.0812	0.0839	0.0951	0.1278	0.0611
AIC	4.51E+04	2.73E+04	2.72E+04	4.42E+04	4.35E+04
BIC	4.54E+04	2.76E+04	2.75E+04	4.45E+04	4.38E+04

*** p<0.01, ** p<0.05, * p<0.10

Category reference classes: Month -January-, Income Perceived -Very Difficult-, Education -Upper secondary-, Age -21-30-, Activity -Paid Work- and Country -Italy-

Table S4 - 2: Models for daily trends before war - 2016-2022

	**Env. Salience**	**Env. Position**	**Sensitivity**
TREND	0.00005***	0.00007***	0.00003***
Big City	0.1448***	0.1435***	-0.0114
Female	0.1458***	0.1713***	0.1096***
Income Perceived			
Difficult	0.0993*	0.0281	-0.1438***
Coping	0.039	-0.0418	-0.2055***
Living comfortably	-0.0155	-0.1453***	-0.1883***
Education			
Less than lower sec.	-0.0994***	-0.1222***	-0.1511***
Lower secondary	-0.1570***	-0.1536***	-0.1092***
Lower upper sec.	-0.2177***	-0.1616***	-0.0840***
Adv. Vocational	-0.0469	-0.0539*	0.0311
Lower tertiary	0.1325***	0.1368***	0.0975***

Higher tertiary	0.2409***	0.2292***	0.1567***
Age			
15-20	-0.0268	-0.0285	-0.0712
31-40	-0.0179	-0.0138	0.1135***
41-50	-0.1155***	-0.0800**	0.1245***
51-60	-0.0620*	-0.0397	0.2370***
61-70	-0.1241***	-0.0823**	0.2877***
71-80	-0.2184***	-0.2023***	0.2505***
81-90	-0.2701***	-0.2284***	0.2624***
Activity			
Education	0.1999***	0.2342***	0.1429***
Un. looking for job	0.1663***	0.1720***	0.0968**
Un. not looking for job	0.1325*	0.0668	-0.0604
Permanent sick or dis.	0.0665	0.0676	0.0957*
Retired	0.0117	0.0266	0.0657**
Military	-0.0779	-0.0667	0.042
Housework	-0.0318	-0.0373	0.0865***
Other	-0.0872	-0.0994	0.3071***
Country			
Belgium	-0.1520***	-0.1477***	-0.0787**
Spain	0.2086***	-0.1222***	0.1146***
the UK	0.0014	0.2079***	-0.1673***
Greece	-0.4654***	-0.6887***	-0.4021***
Norway	0.0855***	-0.1767***	-0.5342***
Poland	-0.4336***	-1.0765***	0.0026
Switzerland	0.2344***	0.0239	0.0463
the Netherlands	0.1490***	-0.3283***	-0.2003***
_cons	-0.1381**	0.1249**	0.0382
N	16476	16476	15805
adj. R-sq	0.0809	0.1272	0.0613
AIC	4.54E+04	4.46E+04	4.38E+04
BIC	4.57E+04	4.49E+04	4.41E+04

*** p<0.01, ** p<0.05, * p<0.10

Category reference classes: Month -January-, Income Perceived -Very Difficult-, Education -Upper secondary-, Age -21-30-, Activity -Paid Work- and Country -Italy-

Appendix S5 Robustness checks

We run many robustness analyses. First, we do a leave-one-out validation by excluding from our main regressions one country at a time (Tables from S5 - 8 to S5 - 16), and then we also run our regressions without IPW weights (Tables S5 - 1 and S5 - 2). Results are not driven by a specific country and are reassuringly similar without the IPW weights. Then, in the event study, we also excluded monthly controls for seasonality (Table S5 - 3). Also this check strongly confirms the results.

This part of the appendix also presents a robustness check where we include two categorical political variables as additional controls: political orientation on a left-right scale and interest in the politics. Below is the figure showing the event study which, as in the main analysis, gives the trend and significance of the period coefficients with respect to the periods before the outbreak of war (see Figure S5 - 1). Again the results appear clear and stable.

The decision to lag energy prices by one month was subjected to a robustness check, considering specifications with no lag (Table S5 - 7) as well as with lags of two (Table S5 - 5) and three months (Table S5 - 6). The specification with a one-month lag was found to perform relatively better, in terms of the significance of the coefficients, the adjusted R-squared, and the informational criteria AIC and BIC.

Finally, as a further check that the effect of the war is not significant on environmental attitudes that are unrelated to policy, we consider in all models an alternative outcome (not available in the ESS for Spain and Poland): the answer to the question "how important to care for nature and environment". Both the event study and the pre-post regression do not show a significant effect and estimations are very close to the case of the Responsibility variable confirming the main results.

Table S5 - 1: Benchmark models without weights

	**Env. Salience**	**Worry**	**Responsibility**	**Env. Position**	**Sensitivity**
Post	-0.0825***	-0.0691**	-0.0221	-0.0779**	-0.0394
Big City	0.1495***	0.0500*	-0.0094	0.1610***	0.0422
Female	0.1419***	0.1908***	0.2174***	0.1917***	0.1394***
Income Perceived					
Difficult	0.0796	0.0164	-0.1807**	0.0728	-0.1965*
Coping	0.0361	-0.1086	-0.2391***	-0.0204	-0.2343**
Living comfortably	0.0479	-0.084	-0.2254**	-0.0464	-0.1795*
Education					
Less than lower sec.	0.0332	-0.0701	-0.0769	-0.0005	-0.2208***
Lower secondary	-0.1375***	-0.0758	-0.1237**	-0.1557***	-0.0313
Lower upper sec.	-0.2371***	-0.0838	-0.0925*	-0.1804***	-0.0951
Adv. Vocational	-0.0525	-0.0109	0.0221	-0.0605	0.0364
Lower tertiary	0.1204***	0.2256***	0.1832***	0.1353***	0.1544***
Higher tertiary	0.2796***	0.3082***	0.1731***	0.2953***	0.2273***
Age					
15-20	0.1083	0.1581*	0.0359	0.1184	-0.1212
31-40	0.0598	0.0377	0.1064*	0.0617	-0.0157
41-50	-0.0851	0.0248	0.1689***	-0.0247	0.0582
51-60	-0.0873	0.0102	0.2183***	-0.0423	0.0448
61-70	-0.1589**	0.1436**	0.2706***	-0.0736	0.1507**
71-80	-0.2250***	0.0016	0.1576**	-0.2343***	0.1137
81-90	-0.3704***	-0.1147	-0.1141	-0.3470***	-0.0006
Activity					
Education	0.1515*	0.0248	0.0503	0.2059**	0.065
Un. looking for job	0.1897**	-0.0045	-0.0835	0.1952**	0.1029
Un. not looking for job	0.0224	0.1131	-0.063	0.0207	0.0715
Permanent sick or dis.	-0.0586	0.034	0.0347	0.058	0.122
Retired	0.0491	0.009	-0.0863*	0.1100**	0.0452
Housework	0.0745	0.0101	-0.0264	0.0554	0.0607
Other	-0.2904	0.0034	-0.0566	-0.3953*	
Country					
Belgium	0.0112	0.0155	0.0268	0.0709	-0.0939*
Spain	0.3626***	0.1958***	0.3786***	0.104	
the UK	0.3554***	0.0205	0.4192***	0.6232***	-0.1250**
Greece	-0.1769***	-0.1133*	-0.3297***	-0.3864***	-0.4061***
Norway	0.2857***	-0.2443***	0.2038***	0.1157**	-0.6177***
Poland	-0.1937***	-0.1477*	0.2360**	-0.5864***	
Switzerland	0.4818***	0.026	0.5087***	0.3191***	-0.1271**

the Netherlands	0.3827***	-0.081	0.2074***	-0.0568	-0.2879***
_cons	-0.3051***	-0.103	-0.2200*	-0.1868*	0.2657**
N	5408	5408	5408	5408	4484
adj. R-sq	0.1026	0.0479	0.0966	0.1227	0.0613
AIC	1.48E+04	1.51E+04	1.48E+04	1.47E+04	1.25E+04
BIC	1.50E+04	1.53E+04	1.51E+04	1.49E+04	1.27E+04

*** p<0.01, ** p<0.05, * p<0.10

Category reference classes: Income Perceived -Very Difficult-, Education -Upper secondary-, Age -21-30-, Activity -Paid Work- and Country -Italy-

Table S5 - 2: Event study without weights

	**Env. Salience**	**Worry**	**Responsibility**	**Env. Position**	**Sensitivity**
Pre-Post Quorters					
+1 (24Feb-23May)	-0.0990***	-0.0824	-0.0084	-0.0963***	-0.0245
-2 (24Aug-23Nov)	-0.0024	-0.0891**	0.021	0.0035	0.0419
-3 (24May-23Aug)	0.0458	-0.0149	0.0502	0.0265	0.0766
Quorters (ESS9-ESS8)	-0.2128	-0.0932	-0.0656	-0.4686	0.1353
Big City	0.1443***	0.0464	-0.0066	0.1545***	0.043
Female	0.1401***	0.2025***	0.2319***	0.1985***	0.1598***
Income Perceived					
Difficult	0.095	0.0383	-0.1827**	0.0951	-0.1941*
Coping	0.0438	-0.0702	-0.2292**	-0.0076	-0.2242**
Living comfortably	0.0523	-0.0602	-0.2180**	-0.0322	-0.1594
Education					
Less than lower sec.	0.0426	-0.0736	-0.0595	0.0033	-0.2013**
Lower secondary	-0.1441***	-0.0696	-0.1119**	-0.1551***	-0.0438
Lower upper sec.	-0.2697***	-0.0952*	-0.0997*	-0.2012***	-0.092
Adv. Vocational	-0.0656	-0.0124	0.0223	-0.0576	0.0011
Lower tertiary	0.1110**	0.2302***	0.1887***	0.1438***	0.1548***
Higher tertiary	0.2358***	0.2931***	0.1653***	0.2617***	0.2334***
Age					
15-20	0.0459	0.1302	-0.0202	0.0812	-0.12
31-40	0.035	0.0158	0.0678	0.0331	-0.0167
41-50	-0.1043*	0.0104	0.1446**	-0.043	0.0555
51-60	-0.0985*	0.0166	0.2197***	-0.0456	0.0639
61-70	-0.1635**	0.1317**	0.2521***	-0.0619	0.1563**
71-80	-0.2468***	-0.0068	0.1295*	-0.2277***	0.133
81-90	-0.3926***	-0.1342	-0.1397	-0.3463***	0.0251
Activity					
Education	0.1431*	0.046	0.0359	0.2017**	0.0893
Un. looking for job	0.2015**	0.0535	-0.0398	0.2183**	0.1065
Un. not looking for job	0.0438	0.1203	-0.0772	0.0134	0.0292
Permanent sick or dis.	-0.0499	0.0699	0.0609	0.0617	0.1443
Retired	0.0477	0.0163	-0.0885*	0.1034**	0.0284
Military service	0.292	0.4077	0.4508	0.5572	0.7595
Housework	0.0745	0.015	-0.0274	0.0531	0.0545
Other	-0.2994*	0.0026	-0.0591	-0.4036*	
Country					
Belgium	-0.0171	0.0421	0.0445	0.0378	-0.097
Spain	0.3731***	0.1950***	0.3829***	0.1237*	
the UK	0.3608***	0.0456	0.3887***	0.6207***	-0.1565**
Greece	-0.1931***	-0.1212*	-0.3286***	-0.4024***	-0.3878***

Norway	0.2998***	-0.2024***	0.1865***	0.1275**	-0.6381***
Poland	-0.1827***	-0.1551*	0.2471**	-0.5690***	
Switzerland	0.4971***	0.0545	0.4805***	0.3279***	-0.1833***
the Netherlands	0.4021***	-0.0496	0.2016***	-0.0422	-0.2949***
_cons	-0.2669**	-0.1133	-0.2190*	-0.1703	0.2313*
N	5026	5026	5026	5026	4116
adj. R-sq	0.1036	0.0492	0.0995	0.1209	0.0619
AIC	1.37E+04	1.40E+04	1.38E+04	1.36E+04	1.15E+04
BIC	1.40E+04	1.43E+04	1.40E+04	1.39E+04	1.17E+04

*** p<0.01, ** p<0.05, * p<0.10

Category reference classes: Income Perceived -Very Difficult-, Education -Upper secondary-, Age -21-30-, Activity -Paid Work-, Country -Italy- and Pre-Post Quorters - -1 (24Nov-23Feb)-

Table S5 - 3: Event study without monthly trends

	**Env. Salience**	**Worry**	**Responsibility**	**Env. Position**	**Sensitivity**
Pre-Post Quorters					
+1 (24Feb-23May)	-0.0951***	-0.1020***	-0.0216	-0.0995***	-0.0365
-2 (24Aug-23Nov)	-0.0184	-0.0641	0.0153	-0.0307	0.0278
-3 (24May-23Aug)	0.0843	0.0261	0.079	0.0602	0.094
Big_City	0.1367***	0.032	-0.0347	0.1796***	0.0509
Female	0.1211***	0.1915***	0.2296***	0.1781***	0.1250***
Income Perceived					
Difficult	0.0546	0.0325	-0.1978*	0.0797	-0.089
Coping	-0.0317	-0.066	-0.2579**	-0.0602	-0.1447
Living comfortably	-0.0273	-0.0987	-0.2664**	-0.0764	-0.1444
Education					
Less than lower sec.	-0.0234	-0.0094	-0.0797	-0.033	-0.2409**
Lower secondary	-0.1210**	-0.051	-0.0708	-0.1115*	-0.041
Lower upper sec.	-0.2318***	-0.0481	-0.0124	-0.1370**	-0.007
Adv. Vocational	-0.0924	-0.0512	0.0536	-0.0332	-0.0277
Lower tertiary	0.0962*	0.1812***	0.1765***	0.1206**	0.1533**
Higher tertiary	0.2401***	0.3134***	0.1981***	0.3105***	0.2309***
Age					
15-20	0.0583	0.0795	-0.0847	0.0923	-0.0837
31-40	0.0362	0.0449	0.0263	0.081	-0.0767
41-50	-0.1073	0.0361	0.1395**	-0.0044	0.0179
51-60	-0.0783	0.0351	0.2403***	0.0135	0.0001
61-70	-0.1364*	0.1825**	0.2691***	-0.009	0.0724
71-80	-0.2379***	0.0181	0.2260**	-0.2027**	0.0946
81-90	-0.3713***	-0.1777	-0.0979	-0.3020***	-0.0489
Activity					
Education	0.1282	0.1103	0.0715	0.2272**	0.0111
Un. looking for job	0.2152**	0.0751	-0.0352	0.2553***	0.1784*
Un. not looking for job	0.0621	0.0105	-0.1635	-0.0372	0.0539
Permanent sick or dis.	-0.1487	-0.037	0.0308	0.0404	0.1181
Retired	0.0497	0.0292	-0.1337**	0.1153*	0.055
Military service	-0.4871	0.5880**	1.0322*	0.2899	1.0056*
Housework	0.0824	0.0677	0.0247	0.0664	0.0836
Other	-0.2507	-0.0889	-0.0706	-0.3803	
Country					
Belgium	-0.0302	0.0782	0.0511	-0.0303	-0.0305
Spain	0.3800***	0.1847***	0.3836***	0.1005	

the UK	0.4390***	0.1009	0.4893***	0.6722***	-0.0561
Greece	-0.2015***	-0.1043	-0.2924***	-0.4217***	-0.4061***
Norway	0.3145***	-0.2506***	0.1778**	0.1094	-0.6990***
Poland	-0.1865***	-0.1973**	0.2287**	-0.6099***	
Switzerland	0.4213***	0.0119	0.4490***	0.2440***	-0.1594**
the Netherlands	0.4030***	-0.0535	0.2094***	-0.0641	-0.2908***
_cons	-0.21	-0.12	-0.2119	-0.1863	0.2384
N	5026	5026	5026	5026	4116
adj. R-sq	0.1064	0.0483	0.0986	0.1316	0.0689
AIC	1.35E+04	1.41E+04	1.39E+04	1.35E+04	1.15E+04
BIC	1.37E+04	1.43E+04	1.42E+04	1.38E+04	1.17E+04

*** p<0.01, ** p<0.05, * p<0.10

Category reference classes: Income Perceived -Very Difficult-, Education -Upper secondary-, Age -21-30-, Activity -Paid Work-, Country -Italy- and Pre-Post Quorters - -1 (24Nov-23Feb)-

Table S5 - 4: Event study without monthly trends and weights

	**Env. Salience**	**Worry**	**Responsibility**	**Env. Position**	**Sensitivity**
Pre-Post Quorters					
+1 (24Feb-23May)	-0.0939***	-0.0973***	-0.0167	-0.0925***	-0.0266
-2 (24Aug-23Nov)	-0.0202	-0.0877**	0.0219	-0.0225	0.035
-3 (24May-23Aug)	0.0407	-0.0245	0.0525	0.0297	0.0893
Big City	0.1444***	0.0465	-0.0066	0.1551***	0.0413
Female	0.1402***	0.2024***	0.2318***	0.1984***	0.1590***
Income Perceived					
Difficult	0.0953	0.0388	-0.1826**	0.0961	-0.1927*
Coping	0.0436	-0.0697	-0.2290**	-0.0071	-0.2228**
Living comfortably	0.0525	-0.0598	-0.2180**	-0.0314	-0.1592
Education					
Less than lower sec.	0.0425	-0.0733	-0.0596	0.0032	-0.2012**
Lower secondary	-0.1444***	-0.0697	-0.1121**	-0.1563***	-0.0438
Lower upper sec.	-0.2704***	-0.095	-0.0995*	-0.2022***	-0.0924
Adv. Vocational	-0.0666	-0.012	0.0224	-0.0589	0.0011
Lower tertiary	0.1107**	0.2305***	0.1886***	0.1423***	0.1550***
Higher tertiary	0.2360***	0.2933***	0.1651***	0.2616***	0.2316***
Age					
15-20	0.0465	0.1311	-0.0204	0.0816	-0.1232
31-40	0.0346	0.016	0.0679	0.0327	-0.0149
41-50	-0.1048*	0.0102	0.1446**	-0.0437	0.0556
51-60	-0.0990*	0.0166	0.2198***	-0.0463	0.0654
61-70	-0.1636**	0.1319**	0.2520***	-0.0631	0.1573**
71-80	-0.2469***	-0.0065	0.1291*	-0.2294***	0.1327
81-90	-0.3930***	-0.1343	-0.1397	-0.3483***	0.0259
Activity					
Education	0.1421*	0.0455	0.036	0.2003**	0.0915
Un. looking for job	0.2012**	0.0528	-0.0395	0.2190**	0.1064
Un. not looking for job	0.044	0.1208	-0.0771	0.0144	0.0281
Permanent sick or dis.	-0.0499	0.0697	0.0606	0.0608	0.1446
Retired	0.0474	0.0159	-0.0882*	0.1040**	0.0286
Military service	0.2784	0.4012	0.4573	0.5555	0.7872
Housework	0.0744	0.0147	-0.0271	0.0533	0.0547
Other	-0.3000*	0.0027	-0.0593	-0.4043*	
Country					

Belgium	-0.0184	0.042	0.0441	0.0378	-0.096
Spain	0.3734***	0.1928***	0.3818***	0.1143*	
the UK	0.3587***	0.0447	0.3881***	0.6177***	-0.1586**
Greece	-0.1903***	-0.1240**	-0.3286***	-0.3967***	-0.3922***
Norway	0.2990***	-0.2036***	0.1857***	0.1251**	-0.6456***
Poland	-0.1812***	-0.1574*	0.2459**	-0.5756***	
Switzerland	0.4930***	0.0522	0.4815***	0.3233***	-0.1774***
the Netherlands	0.4019***	-0.0511	0.2019***	-0.0405	-0.2975***
_cons	-0.2765**	-0.1116	-0.2177*	-0.1808*	0.2328*
N	5026	5026	5026	5026	4116
adj. R-sq	0.1037	0.0494	0.0997	0.1209	0.0619
AIC	1.37E+04	1.40E+04	1.38E+04	1.36E+04	1.15E+04
BIC	1.40E+04	1.43E+04	1.40E+04	1.39E+04	1.17E+04

*** p<0.01, ** p<0.05, * p<0.10

Category reference classes: Income Perceived -Very Difficult-, Education -Upper secondary-, Age -21-30-, Activity -Paid Work-, Country -Italy- and Pre-Post Quorters - -1 (24Nov-23Feb)-

Table S5 - 5: Models with only Energy prices lagged by 2 months

	**Env. Salience**	**Worry**	**Responsibility**	**Env. Position**	**Sensitivity**
Energy price (log - lag2)	-0.0973	-0.0765	-0.0755	-0.0744	-0.0652
Big City	0.1527***	0.0835**	0.0088	0.1574***	0.0577
Female	0.1830***	0.1992***	0.2093***	0.2254***	0.1316***
Income Perceived					
Difficult	0.1361*	0.0318	-0.2191**	0.1453*	-0.1663
Coping	0.1026	-0.0961	-0.2838***	0.0512	-0.2144**
Living comfortably	0.1473*	-0.0834	-0.2611***	0.048	-0.1647
Education					
Less than lower sec.	0.0449	-0.0068	-0.04	0.0183	-0.1935**
Lower secondary	-0.1229**	-0.0405	-0.1318**	-0.1495**	-0.0356
Lower upper sec.	-0.2244***	-0.0362	-0.0279	-0.1660**	0.0163
Adv. Vocational	-0.0516	-0.0025	0.0319	-0.0492	0.0696
Lower tertiary	0.1185**	0.2590***	0.1777***	0.1161**	0.1438**
Higher tertiary	0.2716***	0.3424***	0.1603***	0.2906***	0.2384***
Age					
15-20	0.0571	0.0993	-0.0818	0.0956	-0.1456
31-40	0.0607	0.0145	0.0802	0.1044	-0.0034
41-50	-0.0224	0.0212	0.1288*	0.0283	0.1129
51-60	-0.0107	0.0594	0.2211***	0.0373	0.0652
61-70	-0.1466*	0.1555*	0.2149***	-0.0564	0.1341
71-80	-0.1846**	0.0115	0.1047	-0.1924**	0.1101
81-90	-0.2232*	-0.0261	-0.156	-0.2650**	0.0244
Activity					
Education	0.18	0.1049	0.0183	0.2168**	0.0793
Un. looking for job	0.2586***	0.0789	-0.058	0.2205*	0.2105**
Un. not looking for job	0.0223	0.2834*	0.0858	0.0768	0.0822
Permanent sick or dis.	-0.044	0.0474	0.0577	0.1308	0.1133
Retired	0.0651	-0.0534	-0.0875	0.1169**	0.116
Housework	0.0632	0.0128	-0.0093	0.0563	0.068
Other	-0.2731	0.0985	0.0189	-0.3501*	
Country					
Belgium	0.0033	0.0017	0.0391	0.0988	-0.1226**
Spain	0.3654***	0.1706***	0.3683***	0.1102*	

the UK	0.3554***	0.0315	0.4235***	0.6364***	-0.1171*
Greece	-0.1255*	-0.1664***	-0.3683***	-0.3319***	-0.4321***
Norway	0.1823*	-0.3833***	0.0905	0.04	-0.7555***
Poland	-0.2107***	-0.131	0.2315*	-0.6376***	
Switzerland	0.3465***	-0.046	0.4103***	0.2168**	-0.1495*
the Netherlands	0.3426***	-0.0872	0.1734**	-0.058	-0.3414***
_cons	0.0213	0.2231	0.2479	0.0165	0.5348
N	5408	5408	5408	5408	4484
adj. R-sq	0.0936	0.059	0.1002	0.1222	0.0692
AIC	1.48E+04	1.50E+04	1.47E+04	1.46E+04	1.26E+04
BIC	1.50E+04	1.52E+04	1.49E+04	1.49E+04	1.28E+04

*** p<0.01, ** p<0.05, * p<0.10

Category reference classes: Income Perceived -Very Difficult-, Education -Upper secondary-, Age -21-30-, Activity -Paid Work- and Country -Italy-

Table S5 - 6: Models with only Energy prices lagged by 3 months

	**Env. Salience**	**Worry**	**Responsibility**	**Env. Position**	**Sensitivity**
Energy price (log - lag3)	-0.0980**	-0.0557	-0.0366	-0.0953**	-0.0548
Big City	0.1534***	0.0841**	0.0092	0.1580***	0.0581
Female	0.1832***	0.1997***	0.2101***	0.2253***	0.1316***
Income Perceived					
Difficult	0.1361*	0.0314	-0.2200**	0.1458*	-0.1675
Coping	0.1017	-0.097	-0.2849***	0.0507	-0.2158**
Living comfortably	0.1465*	-0.0843	-0.2622***	0.0476	-0.1663
Education					
Less than lower sec.	0.0438	-0.0079	-0.0411	0.0176	-0.1949**
Lower secondary	-0.1216**	-0.0399	-0.1316**	-0.1481**	-0.0348
Lower upper sec.	-0.2250***	-0.0372	-0.0293	-0.1660**	0.0154
Adv. Vocational	-0.0545	-0.0041	0.0309	-0.0522	0.068
Lower tertiary	0.1183**	0.2589***	0.1776***	0.1159**	0.1438**
Higher tertiary	0.2729***	0.3427***	0.1599***	0.2923***	0.2395***
Age					
15-20	0.0542	0.0984	-0.0814	0.092	-0.146
31-40	0.0624	0.0154	0.0807	0.1062	-0.0015
41-50	-0.0214	0.0215	0.1287*	0.0295	0.1137
51-60	-0.0089	0.0603	0.2215***	0.0391	0.0673
61-70	-0.1447*	0.1561*	0.2148***	-0.054	0.1363
71-80	-0.1831**	0.012	0.1046	-0.1906**	0.1119
81-90	-0.2198*	-0.0251	-0.1564	-0.2607**	0.0278
Activity					
Education	0.1809*	0.1047	0.0174	0.2183**	0.0804
Un. looking for job	0.2619***	0.0816	-0.0553	0.2229*	0.2122**
Un. not looking for job	0.0253	0.2847*	0.0862	0.0801	0.0851
Permanent sick or dis.	-0.0458	0.0469	0.058	0.1286	0.1132
Retired	0.0644	-0.0529	-0.0862	0.1154**	0.1162
Housework	0.0618	0.0121	-0.0096	0.0548	0.0674
Other	-0.2725	0.0992	0.0198	-0.3499*	
Country					
Belgium	-0.014	-0.0036	0.0411	0.0776	-0.1299**
Spain	0.3630***	0.1685***	0.3661***	0.1085*	
the UK	0.3504***	0.0328	0.4293***	0.6276***	-0.1179*
Greece	-0.1320*	-0.1711***	-0.3725***	-0.3374***	-0.4360***

Norway	0.1889**	-0.3563***	0.1360*	0.0242	-0.7399***
Poland	-0.2178***	-0.1275	0.2428*	-0.6517***	
Switzerland	0.3475***	-0.0368	0.4266***	0.2096**	-0.1445*
the Netherlands	0.3425***	-0.0817	0.1837***	-0.0635	-0.3386***
_cons	0.0208	0.1098	0.0382	0.1242	0.4768
N	5408	5408	5408	5408	4484
adj. R-sq	0.0943	0.059	0.0999	0.1232	0.0693
AIC	1.48E+04	1.50E+04	1.47E+04	1.46E+04	1.26E+04
BIC	1.50E+04	1.52E+04	1.49E+04	1.49E+04	1.28E+04

*** p<0.01, ** p<0.05, * p<0.10

Category reference classes: Income Perceived -Very Difficult-, Education -Upper secondary-, Age -21-30-, Activity -Paid Work- and Country -Italy-

Table S5 - 7: Models with only Energy prices not lagged

	**Env. Salience**	**Worry**	**Responsibility**	**Env. Position**	**Sensitivity**
Energy price (log)	-0.092	-0.0829	-0.0659	-0.0872	-0.1108*
Big City	0.1531***	0.0839**	0.0091	0.1577***	0.0582
Female	0.1850***	0.2008***	0.2108***	0.2270***	0.1328***
Income Perceived					
Difficult	0.134	0.0302	-0.2207**	0.1437*	-0.1686
Coping	0.1013	-0.0971	-0.2849***	0.0503	-0.2152**
Living comfortably	0.1460*	-0.0843	-0.2622***	0.0471	-0.1658
Education					
Less than lower sec.	0.042	-0.0092	-0.0422	0.0158	-0.1968**
Lower secondary	-0.1233**	-0.0408	-0.1322**	-0.1498**	-0.0359
Lower upper sec.	-0.2266***	-0.0379	-0.0297	-0.1675**	0.0158
Adv. Vocational	-0.0526	-0.0035	0.0312	-0.0503	0.069
Lower tertiary	0.1165**	0.2573***	0.1763***	0.1143**	0.1419**
Higher tertiary	0.2710***	0.3422***	0.1598***	0.2905***	0.2406***
Age					
15-20	0.0586	0.1002	-0.0805	0.0963	-0.1434
31-40	0.0609	0.0147	0.0803	0.1047	-0.0023
41-50	-0.0227	0.021	0.1285*	0.0282	0.1134
51-60	-0.0097	0.0603	0.2217***	0.0383	0.0684
61-70	-0.1490*	0.1536*	0.2131***	-0.0583	0.1339
71-80	-0.1858**	0.0106	0.1038	-0.1932**	0.1113
81-90	-0.2249*	-0.0271	-0.1574	-0.2657**	0.0279
Activity					
Education	0.178	0.1033	0.0166	0.2154**	0.079
Un. looking for job	0.2605***	0.0802	-0.0564	0.2215*	0.2105**
Un. not looking for job	0.0201	0.2816*	0.0841	0.075	0.082
Permanent sick or dis.	-0.045	0.0462	0.0572	0.1294	0.1114
Retired	0.068	-0.0511	-0.0852	0.1190**	0.1175
Housework	0.0637	0.0131	-0.009	0.0567	0.0687
Other	-0.2735	0.0979	0.0187	-0.3509*	
Country					
Belgium	0.0191	0.0135	0.0517	0.1099	-0.1148*
Spain	0.3547***	0.1613***	0.3605***	0.1007	
the UK	0.3692***	0.0418	0.4345***	0.6460***	-0.1108*
Greece	-0.1294*	-0.1695***	-0.3714***	-0.3349***	-0.4342***
Norway	0.2008**	-0.3789***	0.1102	0.0379	-0.7905***
Poland	-0.2339***	-0.1558	0.2169	-0.6659***	

Switzerland	0.3562***	-0.0418	0.4196***	0.2187**	-0.1592*
the Netherlands	0.3559***	-0.0782	0.1844***	-0.0502	-0.3395***
_cons	0.0063	0.2694	0.2063	0.0982	0.7936**
N	5408	5408	5408	5408	4484
adj. R-sq	0.0934	0.059	0.1	0.1224	0.0698
AIC	1.48E+04	1.50E+04	1.47E+04	1.46E+04	1.26E+04
BIC	1.50E+04	1.52E+04	1.49E+04	1.49E+04	1.28E+04

*** p<0.01, ** p<0.05, * p<0.10

Category reference classes: Income Perceived -Very Difficult-, Education -Upper secondary-, Age -21-30-, Activity -Paid Work- and Country -Italy-

Figure S5 - 1: Event Study including additional controls

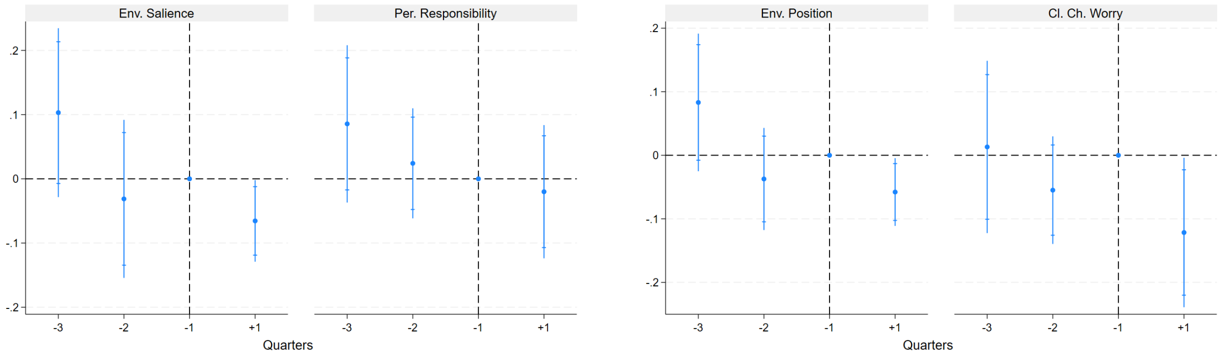

Event graphs come from OLS regressions on 4967 observations on standardized variables over closeness to parties with a strong focus on the environment, personal responsibility towards climate change, closeness to pro-environmental parties and concerns about climate change. Controls include gender, living in a big city, income, education, age-groups, main activity, left-right scale, political interest, a control for monthly trends a and country fixed effects. Independent variables of interest are quarters with 24th November - 23rd February 2022 (-1) as the benchmark. Confidence intervals are at 90% and 95%.

Table S5 - 8: Benchmark models without Belgium

	**Env. Salience**	**Worry**	**Responsibility**	**Env. Position**	**Sensitivity**
Post	-0.1018***	-0.0955***	-0.0325	-0.0833**	-0.067
Big City	0.1419***	0.0978***	0.0102	0.1462***	0.0618
Female	0.1872***	0.2198***	0.2081***	0.2281***	0.1270***
Income Perceived					
Difficult	0.1353	0.0711	-0.1667*	0.1655**	-0.1243
Coping	0.1168	-0.0775	-0.2686***	0.0745	-0.1971*
Living comfortably	0.1482*	-0.0729	-0.2422**	0.0717	-0.1495
Education					
Less than lower sec.	0.0295	-0.0565	-0.0717	-0.0118	-0.2419**
Lower secondary	-0.1418**	-0.0598	-0.1457**	-0.1740***	-0.0664
Lower upper sec.	-0.2668***	-0.075	-0.0495	-0.2004***	-0.0083
Adv. Vocational	-0.0611	-0.0561	0.0343	-0.0727	0.0573
Lower tertiary	0.0977*	0.2170***	0.1848***	0.1138**	0.1147
Higher tertiary	0.1909***	0.2865***	0.1250**	0.2262***	0.2348***
Age					
15-20	0.0015	0.0731	-0.1366	0.0362	-0.1268
31-40	0.0198	-0.0322	0.0753	0.0722	-0.0629

41-50	-0.0692	-0.0116	0.1254*	0.0005	0.0724
51-60	-0.0426	0.0057	0.2438***	0.0255	0.0284
61-70	-0.2230***	0.0572	0.2194***	-0.0976	0.089
71-80	-0.2147**	-0.0964	0.1019	-0.1930**	0.0818
81-90	-0.2565*	-0.1056	-0.1593	-0.2757**	0.0086
Activity					
Education	0.1841	0.0702	0.0421	0.2447**	0.0723
Un. looking for job	0.2553**	0.1106	-0.0326	0.2283*	0.2103**
Un. not looking for job	0.0104	0.3838**	0.1335	0.0675	0.0465
Permanent sick or dis.	0.0526	0.0682	0.0108	0.168	0.0231
Retired	0.0882	0.0224	-0.0582	0.1322**	0.1242
Housework	0.0791	0.0417	0.0206	0.0867	0.0823
Other	-0.2869	0.119	0.0397	-0.3536*	
Country					
the UK	0.3849***	0.061	0.4428***	0.6601***	-0.0957
Spain	0.3849***	0.1787***	0.3743***	0.1243*	
Norway	0.3037***	-0.2851***	0.1827***	0.1297*	-0.6630***
Poland	-0.1639**	-0.1087	0.2600**	-0.6083***	
Switzerland	0.4267***	0.0083	0.4504***	0.2748***	-0.1189
the Netherlands	0.3932***	-0.0461	0.2054***	-0.0272	-0.3138***
Greece	-0.1352*	-0.1845***	-0.3918***	-0.3422***	-0.4326***
_cons	-0.4306***	-0.1023	-0.1818	-0.3296***	0.2686*
N	4844	4844	4844	4844	3921
adj. R-sq	0.095	0.0643	0.1087	0.1303	0.0686
AIC	1.32E+04	1.33E+04	1.31E+04	1.31E+04	1.10E+04
BIC	1.34E+04	1.35E+04	1.33E+04	1.33E+04	1.12E+04

*** p<0.01, ** p<0.05, * p<0.10
Category reference classes: Income Perceived -Very Difficult-, Education -Upper secondary-, Age -21-30-, Activity -Paid Work- and Country -Italy-

Table S5 - 9: Benchmark models without Switzerland

	**Env. Salience**	**Worry**	**Responsibility**	**Env. Position**	**Sensitivity**
Post	-0.0717**	-0.0751**	-0.0249	-0.0696**	-0.0663
Big City	0.1519***	0.0949***	0.0257	0.1664***	0.0965**
Female	0.1717***	0.1619***	0.1810***	0.2041***	0.1106**
Income Perceived					
Difficult	0.1017	0.0427	-0.2270**	0.0984	-0.1951*
Coping	0.0951	-0.0791	-0.2743***	0.0284	-0.1843
Living comfortably	0.0894	-0.0573	-0.2491**	-0.015	-0.1329
Education	0.0338	0.0221	-0.0196	0.0073	-0.1665*
Less than lower sec.	-0.0804	-0.0222	-0.1324**	-0.1202**	-0.0365
Lower secondary	-0.1588**	-0.0265	0.0057	-0.1052	0.0704
Lower upper sec.					
Adv. Vocational	-0.0928	-0.0021	0.0164	-0.0891	0.0065
Lower tertiary	0.0865	0.2837***	0.1932***	0.0943*	0.1562**
Higher tertiary	0.2263***	0.3415***	0.1581***	0.2515***	0.2033***
Age					
15-20	0.1139	0.0635	-0.1471	0.1529	-0.2661
31-40	0.0463	0.0141	0.0859	0.1031	0.036
41-50	-0.0568	0.0105	0.0951	0.0039	0.1075
51-60	-0.0502	0.0476	0.1782**	0.0112	0.0786
61-70	-0.1288*	0.1580*	0.1983**	-0.025	0.1602

71-80	-0.1764**	-0.0074	0.0573	-0.1743*	0.0819
81-90	-0.2730**	-0.046	-0.2621**	-0.3026**	-0.0132
Activity					
Education	0.1193	0.0785	-0.0176	0.1462	0.0781
Un. looking for job	0.2259**	0.0086	-0.1093	0.1521	0.1604*
Un. not looking for job	-0.0154	0.2713*	0.0674	0.033	0.0904
Permanent sick or dis.	-0.0893	0.0475	0.0707	0.1077	0.1138
Retired	0.0098	-0.0643	-0.069	0.0646	0.1138
Housework	0.0497	0.0089	-0.0259	0.0282	0.053
Other	-0.3309*	0.0929	0.0231	-0.4057**	
Country					
the UK	0.4276***	0.0404	0.4287***	0.6977***	-0.1067
Belgium	0.0449	0.0123	0.0474	0.1322*	-0.1056*
Spain	0.4013***	0.1672***	0.3615***	0.1231*	
Norway	0.3281***	-0.3045***	0.1601***	0.1383**	-0.6890***
Poland	-0.1892**	-0.1104	0.2435*	-0.6426***	
the Netherlands	0.4039***	-0.0761	0.1782***	-0.0367	-0.3357***
Greece	-0.1314*	-0.1713***	-0.3726***	-0.3497***	-0.4340***
_cons	-0.3802***	-0.1407	-0.0719	-0.2487**	0.2219
N	4766	4766	4766	4766	3842
adj. R-sq	0.09	0.0605	0.0971	0.1263	0.0721
AIC	1.30E+04	1.32E+04	1.29E+04	1.29E+04	1.08E+04
BIC	1.33E+04	1.34E+04	1.32E+04	1.31E+04	1.10E+04

*** p<0.01, ** p<0.05, * p<0.10

Category reference classes: Income Perceived -Very Difficult-, Education -Upper secondary-, Age -21-30-, Activity -Paid Work- and Country -Italy-

Table S5 - 10: Benchmark models without Spain

	**Env. Salience**	**Worry**	**Responsibility**	**Env. Position**	**Sensitivity**
Post	-0.0926**	-0.0921**	-0.049	-0.0823**	-0.0528
Big City	0.1634***	0.0918**	0.0052	0.1664***	0.0579
Female	0.1865***	0.2076***	0.2027***	0.2340***	0.1316***
Income Perceived					
Difficult	0.0969	0.0401	-0.2510**	0.07	-0.1704
Coping	0.0753	-0.0992	-0.3198***	-0.0186	-0.2088**
Living comfortably	0.0978	-0.0788	-0.2816***	-0.049	-0.1608
Education					
Less than lower sec.	0.0362	-0.0657	-0.0877	0.016	-0.1946**
Lower secondary	-0.1696**	-0.0635	-0.1631**	-0.1933***	-0.0338
Lower upper sec.	-0.2241***	-0.0002	-0.0193	-0.1500**	0.0219
Adv. Vocational	-0.0647	0.0161	0.0471	-0.0539	0.0755
Lower tertiary	0.1310**	0.2925***	0.2055***	0.1211**	0.1472**
Higher tertiary	0.3376***	0.4260***	0.2109***	0.3640***	0.2389***
Age					
15-20	0.1397	0.1292	-0.0986	0.2336	-0.1517
31-40	0.047	-0.007	0.0526	0.1069	-0.0037
41-50	-0.0569	-0.0104	0.0739	0.0114	0.1092
51-60	-0.0348	0.0249	0.1581**	0.0256	0.0638
61-70	-0.1623*	0.1339	0.1701*	-0.0688	0.1334
71-80	-0.1823*	-0.003	0.0495	-0.1826*	0.1102
81-90	-0.2309*	-0.041	-0.1482	-0.2698**	0.0208
Activity					

Education	0.2059	0.154	0.0973	0.2441*	0.0763
Un. looking for job	0.2163*	0.1644	0.0085	0.1639	0.2308**
Un. not looking for job	-0.0556	0.2413	0.0635	-0.0215	0.0899
Permanent sick or dis.	-0.036	0.0201	0.0765	0.1273	0.1258
Retired	0.0617	-0.0439	-0.0812	0.1206*	0.1168
Housework	0.0786	0.0202	0.0108	0.0821	0.0707
Country					
the UK	0.3579***	0.0441	0.4428***	0.6426***	-0.1028
Belgium	0.0069	0.0066	0.043	0.1005	-0.1105*
Norway	0.2724***	-0.3226***	0.1531**	0.1102*	-0.6938***
Poland	-0.2124***	-0.1386	0.2443*	-0.6480***	
Switzerland	0.3631***	-0.0361	0.4383***	0.2280**	-0.1219
the Netherlands	0.3415***	-0.0939	0.1758**	-0.059	-0.3289***
Greece	-0.1350*	-0.1610**	-0.3687***	-0.3456***	-0.4282***
_cons	-0.3730***	-0.1096	-0.0406	-0.2575**	0.2067
N	4653	4653	4653	4653	4484
adj. R-sq	0.105	0.0643	0.1064	0.1459	0.0709
AIC	1.27E+04	1.28E+04	1.26E+04	1.25E+04	1.26E+04
BIC	1.29E+04	1.30E+04	1.28E+04	1.27E+04	1.28E+04

*** p<0.01, ** p<0.05, * p<0.10

Category reference classes: Income Perceived -Very Difficult-, Education -Upper secondary-, Age -21-30-, Activity -Paid Work- and Country -Italy-

Table S5 - 11: Benchmark models without the UK

	**Env. Salience**	**Worry**	**Responsibility**	**Env. Position**	**Sensitivity**
Post	-0.1161***	-0.1134***	-0.0651*	-0.1113***	-0.0831*
Big City	0.1430***	0.0779**	0.0032	0.1463***	0.0616
Female	0.2002***	0.1944***	0.2241***	0.2508***	0.1307***
Income Perceived					
Difficult	0.1316	-0.0051	-0.2249**	0.1436*	-0.2178*
Coping	0.0938	-0.1163	-0.2820***	0.0379	-0.2571**
Living comfortably	0.1634*	-0.1023	-0.2653***	0.0596	-0.2014*
Education					
Less than lower sec.	0.0668	0.0451	-0.0137	0.0341	-0.1278
Lower secondary	-0.1376**	-0.0243	-0.1248**	-0.1660***	-0.0262
Lower upper sec.	-0.2391***	-0.0559	-0.0387	-0.1865**	0.0239
Adv. Vocational	-0.0536	-0.0346	0.0199	-0.0459	0.0439
Lower tertiary	0.0950*	0.2361***	0.1780***	0.0932*	0.1416**
Higher tertiary	0.2497***	0.3224***	0.1522***	0.2728***	0.2335***
Age					
15-20	0.0259	0.0706	-0.1046	0.0773	-0.1681
31-40	0.0627	0.0209	0.0782	0.1068	0.0232
41-50	-0.0039	0.0299	0.1480**	0.0484	0.1318
51-60	0.024	0.0928	0.2545***	0.0693	0.0772
61-70	-0.1072	0.1823**	0.2330***	-0.0172	0.1416
71-80	-0.143	-0.0245	0.0829	-0.1628	0.0988
81-90	-0.1866	-0.0667	-0.2043	-0.2422*	0.0175
Activity					
Education	0.2015*	0.1308	0.0391	0.2374**	0.092
Un. looking for job	0.2707***	0.0968	-0.0549	0.2315*	0.2055*
Un. not looking for job	0.067	0.2595	0.1054	0.1189	0.0808
Permanent sick or dis.	-0.0996	0.0899	0.0502	0.0937	0.1821

Retired	0.0636	-0.0396	-0.0726	0.1239*	0.1615**
Housework	0.0399	0.0227	-0.0349	0.0368	0.0721
Other	-0.2596	0.095	0.0231	-0.3369*	
Country					
Belgium	0.035	0.028	0.0577	0.1217*	-0.1061*
Spain	0.3634***	0.1746***	0.3641***	0.1155*	
Norway	0.2916***	-0.2909***	0.1781***	0.1290**	-0.6841***
Poland	-0.1526**	-0.0852	0.2576**	-0.5866***	
Switzerland	0.3785***	0.0012	0.4476***	0.2498***	-0.1115
the Netherlands	0.3709***	-0.058	0.2010***	-0.0287	-0.3258***
Greece	-0.1166*	-0.1645**	-0.3728***	-0.3233***	-0.4412***
_cons	-0.4514***	-0.1157	-0.114	-0.2973**	0.2608*
N	4891	4891	4891	4891	3969
adj. R-sq	0.0944	0.0628	0.1019	0.0933	0.0731
AIC	1.33E+04	1.35E+04	1.33E+04	1.34E+04	1.11E+04
BIC	1.36E+04	1.37E+04	1.35E+04	1.36E+04	1.13E+04

*** p<0.01, ** p<0.05, * p<0.10

Category reference classes: Income Perceived -Very Difficult-, Education -Upper secondary-, Age -21-30-, Activity -Paid Work- and Country -Italy-

Table S5 - 12: Benchmark models without Greece

	**Env. Salience**	**Worry**	**Responsibility**	**Env. Position**	**Sensitivity**
Post	-0.1155***	-0.0657*	-0.0333	-0.1065***	-0.0402
Big City	0.1895***	0.1099***	0.038	0.1847***	0.0709
Female	0.2177***	0.2025***	0.2251***	0.2516***	0.1441***
Income Perceived					
Difficult	0.17	0.0355	-0.2033	0.2564**	-0.1349
Coping	0.1379	-0.1372	-0.3327**	0.1695	-0.216
Living comfortably	0.1945*	-0.1192	-0.2890**	0.1789	-0.1648
Education					
Less than lower sec.	0.0496	-0.0191	-0.02	0.0348	-0.2144*
Lower secondary	-0.1015	-0.075	-0.1548**	-0.1374**	-0.0489
Lower upper sec.	-0.1991***	-0.0532	-0.0312	-0.1509**	0.0446
Adv. Vocational	-0.0137	-0.0161	0.0073	-0.0334	0.0738
Lower tertiary	0.1826***	0.2535***	0.1309**	0.1535**	0.1713**
Higher tertiary	0.2931***	0.3332***	0.1381**	0.2906***	0.2572***
Age					
15-20	0.0748	0.1228	-0.0796	0.0918	-0.094
31-40	0.0862	0.0388	0.0888	0.1375*	-0.0092
41-50	0.011	0.03	0.1694**	0.0663	0.1087
51-60	-0.0045	0.1094	0.2836***	0.0576	0.0982
61-70	-0.1405*	0.2038**	0.2506***	-0.0517	0.1662
71-80	-0.2170**	0.078	0.1832*	-0.2173**	0.1726
81-90	-0.2109	0.0848	-0.1199	-0.2673**	0.0795
Activity					
Education	0.1814	0.0876	-0.0149	0.2229**	0.0173
Un. looking for job	0.2658**	0.032	-0.1054	0.2147	0.1831
Un. not looking for job	-0.0835	0.2823*	0.181	0.0351	0.3075**
Permanent sick or dis.	-0.0245	0.0459	0.0477	0.1511*	0.1301
Retired	0.1212*	-0.0662	-0.0799	0.1713***	0.1337
Housework	0.0737	0.039	0.101	0.0943	0.1245
Other	-0.2488	0.1026	0.0114	-0.3216*	

Country					
the UK	0.3504***	0.0337	0.4443***	0.6129***	-0.1175*
Belgium	0.0121	0.0203	0.0747	0.1057	-0.1078*
Spain	0.3421***	0.1730***	0.3856***	0.0952	
Norway	0.2626***	-0.2985***	0.1942***	0.1007	-0.6885***
Poland	-0.1904***	-0.0982	0.2981**	-0.5935***	
Switzerland	0.3687***	-0.0167	0.4602***	0.2326***	-0.1272*
the Netherlands	0.3465***	-0.0631	0.2055***	-0.047	-0.3313***
_cons	-0.5987***	-0.161	-0.2448	-0.5613***	0.1285
N	4668	4668	4668	4668	3745
adj. R-sq	0.0937	0.0641	0.0692	0.1143	0.08
AIC	1.28E+04	1.29E+04	1.28E+04	1.27E+04	1.05E+04
BIC	1.30E+04	1.31E+04	1.30E+04	1.29E+04	1.07E+04

*** p<0.01, ** p<0.05, * p<0.10

Category reference classes: Income Perceived -Very Difficult-, Education -Upper secondary-, Age -21-30-, Activity -Paid Work- and Country -Italy-

Table S5 - 13: Benchmark models without Italy

	**Env. Salience**	**Worry**	**Responsibility**	**Env. Position**	**Sensitivity**
Post	-0.1040***	-0.0976***	-0.0408	-0.1026***	-0.052
Big City	0.1696***	0.1020***	0.0269	0.1757***	0.0619
Female	0.2148***	0.2110***	0.2398***	0.2619***	0.1375***
Income Perceived					
Difficult	0.1520*	0.0187	-0.2588***	0.1638**	-0.1588
Coping	0.1016	-0.0928	-0.2965***	0.0537	-0.2131*
Living comfortably	0.1625*	-0.0831	-0.2608***	0.0675	-0.1541
Education	0	0	0	0	0
Less than lower sec.	0.0017	-0.0409	-0.0768	-0.0206	-0.1712*
Lower secondary	-0.1323**	-0.0565	-0.1437**	-0.1676**	-0.0084
Lower upper sec.	-0.2105***	-0.0429	-0.0331	-0.1423*	0.0316
Adv. Vocational	-0.0352	-0.0142	0.0234	-0.0398	0.0966
Lower tertiary	0.1202**	0.2522***	0.1556***	0.1177**	0.1604**
Higher tertiary	0.3048***	0.3483***	0.1559***	0.3233***	0.2631***
Age					
15-20	0.06	0.1105	-0.0964	0.0975	-0.1166
31-40	0.0803	0.0403	0.1162	0.127	0.051
41-50	-0.0085	0.0213	0.1576**	0.0478	0.1492
51-60	0.0231	0.073	0.2291***	0.0739	0.0911
61-70	-0.1358*	0.1953**	0.2311***	-0.022	0.2073**
71-80	-0.2078**	0.0804	0.1451	-0.2002**	0.2034*
81-90	-0.2227*	0.0659	-0.0957	-0.2490*	0.101
Activity	0	0	0	0	0
Education	0.2115*	0.1312	0.016	0.2522**	0.1028
Un. looking for job	0.3181***	0.0866	-0.0512	0.2343*	0.2759**
Un. not looking for job	0.0596	0.3143*	0.1708	0.0922	0.0849
Permanent sick or dis.	-0.0181	0.055	0.0508	0.1572*	0.1503
Retired	0.0725	-0.0831	-0.0895	0.1287**	0.1011
Housework	0.0722	-0.0033	-0.0241	0.0458	0.0529
Other	-0.2717	0.1058	0.0201	-0.3401*	
Country					
Belgium	-0.3688***	-0.0285	-0.3777***	-0.5460***	0.0042
Spain	-0.0234	0.1255**	-0.0667	-0.5539***	

Norway	-0.1036**	-0.3415***	-0.2630***	-0.5449***	-0.5646***
Poland	-0.5797***	-0.1584*	-0.1857	-1.2839***	
Switzerland	-0.0074	-0.0591	-0.0003	-0.4229***	-0.0131
the Netherlands	-0.0209	-0.1152	-0.2487***	-0.7089***	-0.2145***
Greece	-0.5210***	-0.2143***	-0.8016***	-0.9944***	-0.3138***
_cons	-0.146	0.2548*	-0.1256	0.2481**	0.0495
N	4896	4896	4896	4896	3974
adj. R-sq	0.1072	0.1053	0.0657	0.1435	0.0675
AIC	1.33E+04	1.33E+04	1.35E+04	1.31E+04	1.11E+04
BIC	1.35E+04	1.35E+04	1.37E+04	1.34E+04	1.13E+04

*** p<0.01, ** p<0.05, * p<0.10

Category reference classes: Income Perceived -Very Difficult-, Education -Upper secondary-, Age -21-30-, Activity -Paid Work- and Country - England-

Table S5 - 14: Benchmark models without the Netherlands

	**Env. Salience**	**Worry**	**Responsibility**	**Env. Position**	**Sensitivity**
Post	-0.0967***	-0.0975***	-0.0422	-0.1019***	-0.0429
Big City	0.1292***	0.0661*	-0.0064	0.1266***	0.029
Female	0.1341***	0.2165***	0.2046***	0.1793***	0.1046**
Income Perceived					
Difficult	0.1438*	-0.0239	-0.2726***	0.1143	-0.2196**
Coping	0.0816	-0.1383	-0.3242***	0.011	-0.2725**
Living comfortably	0.1617*	-0.1308	-0.3266***	0.0346	-0.2358**
Education					
Less than lower sec.	0.0929	0.0594	-0.0087	0.0765	-0.2159**
Lower secondary	-0.0805	0.0038	-0.1022*	-0.1054*	-0.0232
Lower upper sec.	-0.2226***	0.0093	-0.0193	-0.1571**	0.0127
Adv. Vocational	-0.0422	0.0318	0.0534	-0.0198	0.0942
Lower tertiary	0.1235**	0.2720***	0.1874***	0.1188**	0.1848***
Higher tertiary	0.2814***	0.3440***	0.1661***	0.3018***	0.2775***
Age					
15-20	0.0678	0.1511	0.0486	0.0829	0.0103
31-40	0.0725	0.0082	0.0911	0.0898	0.017
41-50	-0.0003	0.0132	0.1361*	0.0192	0.123
51-60	-0.0281	0.0117	0.2211***	-0.0198	0.0281
61-70	-0.1429*	0.1253	0.2179***	-0.0972	0.0819
71-80	-0.2116**	-0.0478	0.087	-0.2716***	0.063
81-90	-0.2579**	-0.1235	-0.1312	-0.3802***	-0.0263
Activity					
Education	0.1474	0.0522	0.0149	0.1356	0.0448
Un. looking for job	0.2499**	0.062	-0.0825	0.2058*	0.1741
Un. not looking for job	0.0221	0.2915*	0.0741	0.0838	0.0548
Permanent sick or dis.	-0.0281	0.0508	0.0915	0.1741*	0.1259
Retired	0.0585	-0.0806	-0.1103*	0.0982	0.1353*
Housework	0.0243	0.001	-0.0302	0.0172	0.0777
Other	-0.2956	0.0702	-0.0053	-0.3885**	
Country					
the UK	0.3806***	0.0541	0.4343***	0.6712***	-0.1023
Belgium	0.0253	0.0239	0.0531	0.1181	-0.1245**
Spain	0.3622***	0.1598***	0.3489***	0.1063	
Norway	0.2929***	-0.3016***	0.1686***	0.1227*	-0.6846***
Poland	-0.1691**	-0.1074	0.2476*	-0.6187***	

Switzerland	0.3752***	-0.0248	0.4276***	0.2361**	-0.1376*
Greece	-0.1148	-0.1610**	-0.3661***	-0.3197***	-0.4326***
_cons	-0.4038***	-0.0822	-0.0792	-0.2616**	0.2930*
N	4770	4770	4770	4770	3848
adj. R-sq	0.0901	0.0627	0.1032	0.1264	0.0744
AIC	1.31E+04	1.32E+04	1.29E+04	1.29E+04	1.07E+04
BIC	1.33E+04	1.34E+04	1.31E+04	1.31E+04	1.09E+04

*** p<0.01, ** p<0.05, * p<0.10

Category reference classes: Income Perceived -Very Difficult-, Education -Upper secondary-, Age -21-30-, Activity -Paid Work- and Country -Italy-

Table S5 - 15: Benchmark models without Norway

	**Env. Salience**	**Worry**	**Responsibility**	**Env. Position**	**Sensitivity**
Post	-0.0948***	-0.0641*	-0.0318	-0.0864**	-0.0137
Big City	0.1490***	0.0358	-0.0284	0.1662***	0.0272
Female	0.1707***	0.1933***	0.2129***	0.2091***	0.1823***
Income Perceived					
Difficult	0.1361*	0.0297	-0.2046**	0.1468*	-0.1252
Coping	0.1026	-0.1033	-0.2664***	0.0597	-0.1907*
Living comfortably	0.1366*	-0.0954	-0.2591***	0.0407	-0.1476
Education					
Less than lower sec.	0.0271	-0.0249	-0.0591	-0.0057	-0.2081**
Lower secondary	-0.1377**	-0.0374	-0.1162*	-0.1489**	-0.0479
Lower upper sec.	-0.2651***	-0.0502	-0.0702	-0.2246***	-0.0938
Adv. Vocational	-0.0478	0.0654	0.0251	-0.0288	0.1292
Lower tertiary	0.1186**	0.2637***	0.1663***	0.1074**	0.0663
Higher tertiary	0.2752***	0.3336***	0.1377**	0.2883***	0.1835***
Age					
15-20	0.0157	0.0588	-0.0432	0.017	-0.2949
31-40	0.0411	-0.0129	-0.0024	0.0498	-0.0978
41-50	-0.0337	0.0447	0.0978	-0.0164	0.0841
51-60	0.0019	0.0796	0.1816**	0.0231	0.0636
61-70	-0.1497*	0.1602*	0.1890**	-0.0821	0.09
71-80	-0.1528	0.0826	0.1088	-0.1672*	0.0557
81-90	-0.1743	0.013	-0.1266	-0.1661	-0.0039
Activity					
Education	0.132	0.1013	-0.0487	0.2040*	0.178
Un. looking for job	0.2560**	0.0831	-0.0416	0.2366*	0.2305**
Un. not looking for job	0.1494	0.1634	-0.1614	0.1667	-0.082
Permanent sick or dis.	-0.14	0.0065	0.0579	0.041	0.0551
Retired	0.0464	-0.0648	-0.1345*	0.0831	0.0548
Housework	0.0761	-0.0224	-0.0792	0.0493	0.0208
Other	-0.2478	0.0858	-0.0042	-0.3190*	
Country					
the UK	0.3542***	0.0449	0.4413***	0.6211***	-0.0829
Belgium	0.025	0.018	0.0571	0.1127	-0.0955
Spain	0.3461***	0.1712***	0.3669***	0.1047*	
Poland	-0.1704**	-0.0876	0.2588**	-0.5903***	
Switzerland	0.3753***	-0.0185	0.4446***	0.2493***	-0.1078
the Netherlands	0.3597***	-0.0544	0.2112***	-0.0259	-0.2933***
Greece	-0.1335*	-0.1557**	-0.3512***	-0.3316***	-0.4387***
_cons	-0.3979***	-0.1787	-0.0791	-0.2813**	0.1572

N	4527	4527	4527	4527	3605
adj. R-sq	0.0923	0.0422	0.1043	0.1246	0.0441
AIC	1.24E+04	1.26E+04	1.23E+04	1.22E+04	1.02E+04
BIC	1.26E+04	1.28E+04	1.26E+04	1.25E+04	1.03E+04

*** p<0.01, ** p<0.05, * p<0.10

Category reference classes: Income Perceived -Very Difficult-, Education -Upper secondary-, Age -21-30-, Activity -Paid Work- and Country -Italy-

Table S5 - 16: Benchmark models without Poland

	**Env. Salience**	**Worry**	**Responsibility**	**Env. Position**	**Sensitivity**
Post	-0.0972***	-0.0927***	-0.0424	-0.0897***	-0.0528
Big City	0.1540***	0.0802**	0.0061	0.1515***	0.058
Female	0.1823***	0.1962***	0.2008***	0.2202***	0.1310***
Income Perceived					
Difficult	0.1408*	0.0436	-0.1961**	0.1579**	-0.1635
Coping	0.1082	-0.0694	-0.2398***	0.0545	-0.2115**
Living comfortably	0.1493*	-0.0614	-0.2268**	0.0425	-0.1665
Education					
Less than lower sec.	0.0481	-0.0093	-0.0251	0.02	-0.1950**
Lower secondary	-0.1204*	-0.0479	-0.1240**	-0.1375**	-0.034
Lower upper sec.	-0.2301***	-0.0549	-0.0242	-0.1729**	0.015
Adv. Vocational	-0.0498	-0.0118	0.0458	-0.0549	0.0706
Lower tertiary	0.1228**	0.2429***	0.1839***	0.1323***	0.1443**
Higher tertiary	0.2772***	0.3272***	0.1758***	0.2935***	0.2397***
Age					
15-20	0.0655	0.1263	-0.0539	0.1028	-0.1345
31-40	0.0687	0.0457	0.1207	0.1274	-0.0006
41-50	-0.0151	0.0464	0.1452**	0.047	0.1132
51-60	-0.0079	0.0808	0.2338***	0.0523	0.069
61-70	-0.1516*	0.1715**	0.2152***	-0.0622	0.1361
71-80	-0.1842**	0.0271	0.1165	-0.1834**	0.1126
81-90	-0.2266*	-0.0249	-0.1789	-0.2553**	0.0278
Activity					
Education	0.1749	0.1043	0.0214	0.2230**	0.0801
Un. looking for job	0.3082***	0.0901	-0.0356	0.3350***	0.2209**
Un. not looking for job	0.0312	0.2960*	0.1026	0.1025	0.0783
Permanent sick or dis.	-0.0419	0.0443	0.0728	0.1298	0.1131
Retired	0.0734	-0.0553	-0.078	0.1323**	0.1181
Housework	0.0651	0.0082	-0.0024	0.0641	0.0693
Other	-0.2767	0.1109	0.0205	-0.3534*	
Country					
the UK	0.3702***	0.0507	0.4390***	0.6584***	-0.1041
Belgium	0.023	0.0235	0.0545	0.1169	-0.1065*
Spain	0.3574***	0.1717***	0.3653***	0.1084*	
Norway	0.2874***	-0.2917***	0.1727***	0.1300**	-0.6820***
Switzerland	0.3847***	-0.0104	0.4398***	0.2569***	-0.1236
the Netherlands	0.3663***	-0.0593	0.1950***	-0.0316	-0.3204***
Greece	-0.1302*	-0.1602**	-0.3637***	-0.3422***	-0.4358***
_cons	-0.4714***	-0.1747	-0.1904	-0.3776***	0.2063
N	5247	5247	5247	5247	4484
adj. R-sq	0.0942	0.0599	0.102	0.1169	0.0691
AIC	1.43E+04	1.45E+04	1.42E+04	1.42E+04	1.26E+04

BIC	1.46E+04	1.48E+04	1.45E+04	1.45E+04	1.28E+04

*** p<0.01, ** p<0.05, * p<0.10

Category reference classes: Income Perceived -Very Difficult-, Education -Upper secondary-, Age -21-30-, Activity -Paid Work- and Country -Italy-
